# An integrative microenvironment approach for laryngeal carcinoma: the role of immune/methylation/autophagy signatures on disease clinical prognosis and single-cell genotypes

**DOI:** 10.7150/jca.58076

**Published:** 2021-05-13

**Authors:** Xueran Kang, Yisheng Chen, Bin Yi, Xiaojun Yan, Chenyan Jiang, Bin Chen, Lixing Lu, Yuxing Sun, Runjie Shi

**Affiliations:** 1 Department of Otolaryngology-Head and Neck Surgery, Shanghai ninth people's Hospital, Shanghai Jiao Tong University School of Medicine; Ear Institute, Shanghai JiaoTong University School of Medicine; Shanghai Key Laboratory of Translational Medicine on Ear and Nose diseases, Shanghai, China.; 2 Department of Orthopedics, Shanghai General Hospital, Shanghai Jiao Tong University School of Medicine, Shanghai, China.

**Keywords:** methylation, autophagy, immune infiltration, laryngocarcinoma, immunotherapy, ceRNA network

## Abstract

The effects of methylation/autophagy-related genes (MARGs) and immune infiltration in the tumor microenvironment on the prognosis of laryngeal cancer were comprehensively explored in this study. Survival analysis screened out 126 MARGs and 10 immune cells potentially associated with the prognosis of laryngeal carcinoma. Cox and lasso regression analyses were then used to select 8 MARGs (CAPN10, DAPK2, MBTPS2, ST13, CFLAR, FADD, PEX14 and TSC2) and 2 immune cells (Eosinophil and Mast cell) to obtain the prognostic risk scoring system (pRS). The pRS was used to establish a risk prediction model for the prognosis of laryngeal cancer. The predictive ability of the prediction model was evaluated by GEO datasets and our clinical samples. Further analysis revealed that pRS is highly associated with single nucleotide polymorphism (SNP), copy number variation (CNV), immune checkpoint blockade (ICB) therapy and tumor microenvironment. Moreover, the screened pRS-related ceRNA network and circ_0002951/miR-548k/HAS2 pathway provide potential therapeutic targets and biomarkers of laryngocarcinoma. Based on the clustering results of pRS-related genes, single cells were then genotyped and revealed by integrated scRNA-seq in laryngeal cancer samples. Fibroblasts were found enriched in high risk cell clusters at the scRNA-seq level. Fibroblast-related ligand-receptor interactions were then exposed and a neural network-based deep learning model based on these pRS-related hub gene signatures was also established with a high accuracy in cell type prediction. In conclusion, the combination of single-cell and transcriptome laryngeal carcinoma landscape analyses can investigate the link between the tumor microenvironmental and prognostic characteristics.

## Introduction

Based on the current understanding, head and neck cancer (HNC) is the 7^th^ most cause of cancer-related deaths globally 1. About 30% of cases described as head and neck cancers are laryngeal cancer. Although the understanding of the molecular biology of laryngeal cancer has been deeply elaborated recently, the survival rate of patients with laryngeal cancer has not changed. So that requires us to screen out molecular biomarkers related to the prognosis of laryngeal cancer to guide individual treatment and improve the survival rate of patients with laryngeal cancer. In the future, biomarkers may become the gold standard in terms of the choice of patient treatment 2.

Autophagy is a progressive degradation process executed by lysosomes and stringently regulated by a series of autophagy-related genes (ARGS). Notably, autophagy is key in maintaining cytoplasmic and genomic integrity and participate in the development of cancer-related abnormalities 3. However, in different tumor types and stages 4, autophagy can either exert inducer or inhibitory roles. For instance, autophagy eliminates damaged organelles and DNA to maintain normal cell structure and metabolic stability, which inhibits the occurrence of cancer cells 5. In tumor progression, autophagy actively degrades more proteins and organelles, enriching tumors with nutrients that promote their proliferation and invasion 6. m^6^A-RNA methylation is among the most critical internal modification in eukaryotic cells. A wealth of evidence indicates that the expression and genetic changes of m^6^A regulatory factors are related to tumor malignant progression and abnormal immune regulation 7-9. A study by Bo Zhang revealed that the m^6^A modification pattern in individual tumors can predict tumor stage, subtype, TME matrix activity, genetic variation and patient prognosis 10.

Additionally, tumor cell immune cells (TIIC), including B cells, dendritic cells, macrophages, neutrophils, T cells, monocytes, and mast cells, participate in the progression of cancer 11-14. Evaluating the lymphocyte infiltration degree of the tumor has been proved to be a critical supplementary indicator of the TNM stage, recurrence and mortality prediction system 15-17. Other than lymphocytes, tumors pose a variety of non-lymphocyte immune cells, 18, 19 which are believed to exhibit a particular effect on the prognosis of different stages of cancers 20.

Recent studies have revealed that m^6^A methylation, immune cell infiltration and autophagy coordinatively play a role in tumor microenvironment 21. m^6^A modification can impact the stability of autophagy-related gene transcripts 21, whereas, m^6^A methylation-related proteins can reduce the presentation of tumor antigens and antigen-specific CD8 T cells anti-tumor response, leading to tumor immune escape and cancer development 22-24. Although a highly coordinated interaction between autophagy, m^6^A methylation-related genes and immune infiltration exist 9, their comprehensive application as specific markers for the analysis of tumor microenvironment and predicting the prognosis of laryngeal cancer has not been described. In this study, we comprehensively analyzed the relationship between the degree of autophagy, methylation, and immune infiltration in tumor tissue and prognosis, aiming at identifying an ideal and accurate tumor prognostic markers to uncover new tumor treatment targets 25, 26.

Single-cell transcriptomic analysis is a powerful method that has emerged recently to explore the tumor microenvironment, enabling the analysis of cellular states and transitions from a single-cell perspective, thereby exploring integrated information across the genome of a tumor sample 27. Recently developed methods for analyzing single-cell data provide many effective ways to explore molecular changes at the cellular level 28. Sorting tumor cells by differentiation trajectories helps us understand the subset of tumor cells and their associated mechanisms of malignant translocation 29. In addition, CellPhoneDB database (www.CellPhoneDB.org/) can be used to predict cell type-specific ligand-receptor complexes 30.

In this study, a systematic analysis of the relationship among immune/methylation/autophagy signatures, laryngeal carcinoma prognosis and the tumor microenvironment was performed. The integrative microenvironment approach was conducted at both macro and micro-levels in order to find a validated prognostic scoring system and new targets for the treatment of laryngeal carcinoma.

## Materials and Methods

### Data retrieval and processing

We downloaded the RNA-seq data of the laryngeal carcinoma queue from the TCGA database and standardized the combined data using the “affy” and “simpleaffy” packages of R software (version 3.5.2) 31. Additional data on simple nucleotide variation (SNP) and copy number variation (CNV) of the laryngeal carcinoma queue were downloaded for further analysis. GSE27020-GPL96 ([HG-U133A] Affymetrix Human Genome U133A Array) mRNA expression array dataset with high data quality and the large sample size was standardized by the “normalize between array” function of the “LIMMA” R package in the Bioconductor project 31. The scRNA-seq data (accession number GSE150321) in laryngeal carcinoma were obtained from the Gene Expression Omnibus (GEO, http://www.ncbi.nlm.nih.gov/geo/) (Table 1). All original platform files were saved.

### Study participants

We used data from clinical samples to further validate the prediction capability of the model. The data were obtained from patients undergoing laryngeal cancer surgery at the Ninth People's Hospital affiliated to Shanghai Jiaotong University. We collected patient-related clinical data via telephone and outpatient follow-up. All subjects signed informed consent. The study was conducted in accordance with the guidelines of the Declaration of Helsinki and was approved by the Ethics Review Committee of the Ninth People's Hospital Affiliated to Shanghai Jiaotong University (approval no. 2017-323-T243).

### CIBERSORT estimation and extraction of m^6^A methylated- and autophagy- associated genes

We uploaded the standardized annotated gene expression datasets to the CIBERSORT website (http://cibersort.stanford.edu/) and initiated the algorithm using the LM22 signature and 1000 permutations 32. Cases with a CIBERSORT output of p <0.05 were subjected to further analysis 33. A total of 222 sites of autophagy-related genes were extracted from HADb (Human Autophagy Database, http://www.autophagy.lu/). According to previously published reviews 34, we collected 16 m^6^A RNA methylation regulators (ALKBH5, WTAP, KIAA1429, METTL3, METTL14, FTO, RBM15, METTL16, YTHDC1, YTHDC2, YTHDF1, YTHDF2, YTHDF3, HNRNPA2B1, ZC3H13, and HNPC) with available expression data in the TCGA datasets. We adopted web-based tools (http://molpath.charite.de/cutoff/) to calculate the entire queue and get the best cut-off value 35.

### Identification and screening of differentially immune infiltrating cells, methylated and autophagy associated genes

First, we analyzed the expression levels of LM22, methylated, and autophagy associated genes of the selected cases using the survfit function and Kaplan-Meier survival analysis in the “survival” software package of R software. Then, 126 genes and 10 types of immune cells related to prognosis were selected. Using univariate Cox regression and multivariate Cox regression analyses, we further screened out 16 genes and 2 immune cells highly correlated with prognosis. Thereafter, the least absolute shrinkage and selection operator (LASSO) and Cox method were adopted to reduce the dimensions, we selected immune cells, methylated, and autophagy associated genes with the most significant risk of death to establish a Cox prognosis model 36. The laryngeal cancer death risk nomogram prediction model was established and verified by the receiver operating characteristic curve (ROC) 37, 38. The performance of the model was assessed by the C index 39. Decision curve analysis was used to evaluate the clinical utility of the nomogram 40. Lastly, we calculated the net benefit according to a previously published study as previous report 41, 42.

### Quantitative real-time PCR (qPCR)

Total mRNA was extracted from cell cultures using the Mini-BEST Universal RNA Extraction kit (TaKaRa, Kyoto, Japan), followed by cDNA synthesis using the Prime-Script RT Master Mix (TaKaRa). qPCR assays were performed using SYBR Green Master Mix (TaKaRa) with PCR LightCycler480 (Roche Diagnostics, Basel, Switzerland).

### Immune cellular fraction estimates and tumor purity analysis

The relative proportions of immune cell types in the leukocyte compartment were estimated using the gene set introduced by Gabriela et al. as described earlier 43, 44. The single sample Gene Set Enrichment Analysis (ssGSEA) was used to score the enrichment of immune cell type meta genes in the given sample using the TPM data of TCGA laryngeal cancer RNA sequence as input, as described in the GSVA package of the R software 45.

The data were then z-scored based on predictions of immune cell infiltration and enrichment. In addition, this study used unsupervised cluster analysis to identify different modification patterns in immune cells and to classify the samples for further study. The clustering algorithm determined the number and stability of clusters 46. The above steps are performed using the consunseClusterPlus package and repeated 1000 times to ensure stability of the classification 47. Finally, the tumor purity score was estimated by the estimation method as described previously 48, 49.

### Predicting pRS-related ceRNA network

All pRS-related mRNAs, miRNAs were selected using “LIMMA” R package from the Bioconductor project 50. The miRWalk3.0 database (http://mirwalk.umm.uni-heidelberg.de/) composed of 10 databases (PITA, Targetscan, PICTAR5, miRanda, miRDB, miRWalk, RNA22, RNAhybrid, PICTAR4, and DIANAmT), and the miRTarBase was used to reveal correlations between pRS-related mRNAs and pRS-related miRNAs 51, 52. All circRNAs set were downloaded form GSE117001 database (https://www.ncbi.nlm.nih.gov/geo/query/acc.cgi?acc=GSE117001). And “LIMMA” R package was also used to identify laryngocarcinoma-related circRNAs by selecting circRNAs different in expression between laryngeal cancer and normal samples [Adj. p value < 0.05 and log fold change (FC) > 2 were considered as statistically significant]. And Circular RNA Interactome (https://circinteractome.nia.nih.gov/index.html) was used to reveal correlation between laryngocarcinoma-related circRNAs and pRS-related miRNAs. 53 Survival analysis was then used to determine pRS-related mRNAs, pRS-related miRNAs and laryngocarcinoma-related circRNAs. To explore the potential pRS-related ceRNA regulatory network in patients with laryngeal cancer, the correlation between pRS-related mRNAs and laryngocarcinoma-related circRNAs was measured by Pearson correlation analysis. The structural patterns of key laryngocarcinoma-related circRNAs were found from CSCD database (https://gb.whu.edu.cn/CSCD/). A pRS-related ceRNA network was illustrated by using Cytoscape (3.8.0) 54. And TIDE (http://tide.dfci.harvard.edu/setquery/) was used to compare new biomarkers with existed biomarkers 55, 56. Furthermore, the immunofluorescent analysis of key pRS-related mRNAs was obtained from THE HUMAN PROTEIN ATLAS 57.

### GSVA and GSEA enrichment analysis

Here, we performed GSVA- and GSEA- enrichment analysis to reveal the biological processes and pathways associated with pRS. In most cases, GSVA is used to evaluate pathway variation and biological processes 45. The gene sets of “c2.cp.kegg.v6.2.-symbols” and “c5.all.v6.2.symbols” were downloaded from MSigDB database for GSVA analysis. Gene set enrichment analysis (GSEA) was used to assess the potential pRS related mechanisms using the project of JAVA (http://software.broadinstitute.org/gsea/index.jsp) 58. The threshold for statistical significance was set at P < 0.05.

### Single‑cell RNA‑seq analysis

Transcriptome profles from GSE150321 were used to perform the single‑cell RNA‑seq analysis with the “Seurat” package 59. The UMAP method is also used for nonlinear dimensionality reduction, followed by the “Seurat” package to discover marker genes between clusters 60. The singleR package was then used for cell cluster annotation based on the composition of the marker genes, which was then corrected using the CellMarker database 61, 62. Single-cell pseudotime trajectories of the laryngeal cancer scRNA-seq data were constructed by the monocle 2 algorithm 63. For data interpretation of single-cell pseudotime trajectories, cells on different branches have different differentiation characteristics. CellPhoneDB database functionality was used to perform cell-to-cell interaction analysis, and cell-to-cell interactions with p-values <0.01 were considered statistically significant 30.

### Neural network-based deep learning framework construction

We constructed a neural network using Python (version 3.7) software's PyTorch framework to predict cell types in single-cell data from screened pRS-related genes 64. All cells were randomly assigned to the training or test groups in a 7:3 ratio. A random gradient descent method was used for the mechanical learning optimizer, while the learning rate was set to 0.001. reLU was set as the activation function. During training, the dropout rate was set to 0.2 for each level.

### Statistical analysis

All statistical data were analyzed using GraphPad Prism (version 7.0) software and R software (version 3.6.1). The Kaplan-Meier method was used to calculate the overall survival rate, as highlighted in the previous research method 65. Conditional Survival (CS) was defined as the probability that the patient would survive for “y” years because the patient survived for “x” years. CS was calculated as CS_(x|y)_ = S_(x+y)_/S_(x)_, and S (x) represented the X-year overall survival estimated using the Kaplan-Meier method 65-69. Statistical significance between groups was determined using either one-way analysis of variance or two-tailed t-test. For correlation analysis, we used Pearson's correlation. *P <0.05 was considered to be statistically significant.

## Results

### Identification and screening of differentially immune infiltrating cells, methylated and autophagy associated genes

**Figure 1:**
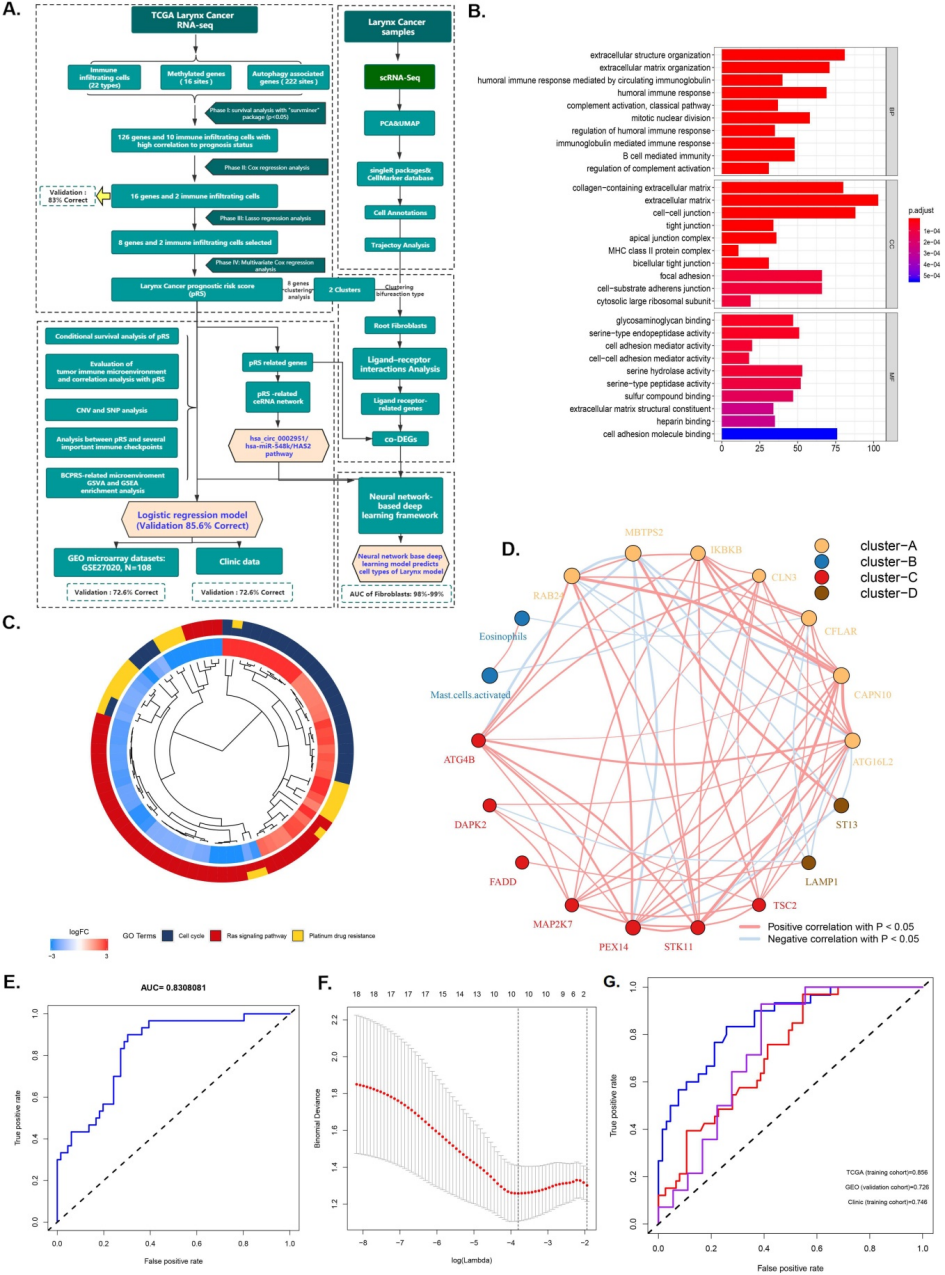
** Identification and screening of differentially immune infiltrating cells, methylated, and autophagy associated genes. A.** Schematic diagram of the study process. The 96 laryngeal carcinoma samples were retrieved from the TCGA database, including 30 dead patients and 66 surviving patients. Survival analysis identified 126 genes and 10 immune cells that are associated with laryngeal cancer prognosis. Subsequent Cox regression identified 16 genes and 2 immune cells related to the prognosis of laryngeal cancer, at 83% corresponding verification score. The gene regulatory network outlines the interaction between methylated and autophagy associated genes and their impact on the prognosis of patients with laryngeal cancer. Further screening using lasso analysis established a predictive model containing 8 genes and 2 best characteristics of immune cells. Finally, a nomogram predictive model containing the best 10 features was constructed, and the predictive model was tested using the GSE27020 set and clinical set. **B.** Functional annotation of 126 sites methylated and autophagy associated genes was completed via GO enrichment analysis. The adjusted P-value of enriched genes is represented by the color depth. **C.** KEGG Cluster: The inner ring shows the color-coded logFC and the outer ring is the enriched KEGG pathway. **D.** The AUC curve showing the predictive ability of the 16 genes and 2 immune cells screened via multifactor COX regression. **E.** Best features selection using the LASSO regression model. The selection of the optimal parameters (lambda) in the LASSO model applied the minimum criterion of 5-fold cross-validation. The dashed line was plotted at the best value using the minimum criterion and the 1se (standard error) of the minimum criterion. **F.** The AUC curve of the laryngeal cancer prognosis prediction model demonstrates the accuracy of the prediction model.

The research plan is shown in Figure 1A. A total of 96 laryngeal carcinoma samples were retrieved from the TCGA database, including 30 dead patients and 66 surviving patients. Survival analysis identified 126 genes and 10 immune cells associated with the prognosis of laryngeal cancer. These 126 laryngeal cancer prognosis-related genes were enriched in BPs (humoral immune response, extracellular structure/matrix organization), CCs (extracellular matrix, cell-cell junction, focal adhesion, and cell-substrate adherens junction), and MFs (cell adhesion molecule binding, serine-type endopeptidase activity, and serine hydrolase activity) (Figure 1C). Pathway analysis showed that those 126 genes were prima involved in the cell cycle, ras signaling pathways as well as in platinum drug resistance (P <0.05; Figure 1D). Further, Cox regression analysis identified 16 genes and 2 immune cells related to the prognosis of laryngeal cancer, with an 83% verification score (Figure 1E). The gene regulatory network described the interaction of methylated with autophagy associated genes and their impact on the prognosis of laryngeal cancer patients (Figure 1B). After further screening using Lasso regression analysis, we established a prediction set containing the best characteristics of 8 genes and 2 immune cells (Figure 1F), (72.6% correction in GSE27020 set, and 74.6% correction in the clinical set).

### The prognostic risk score (pRS) for predicting laryngocarcinoma

**Figure 2:**
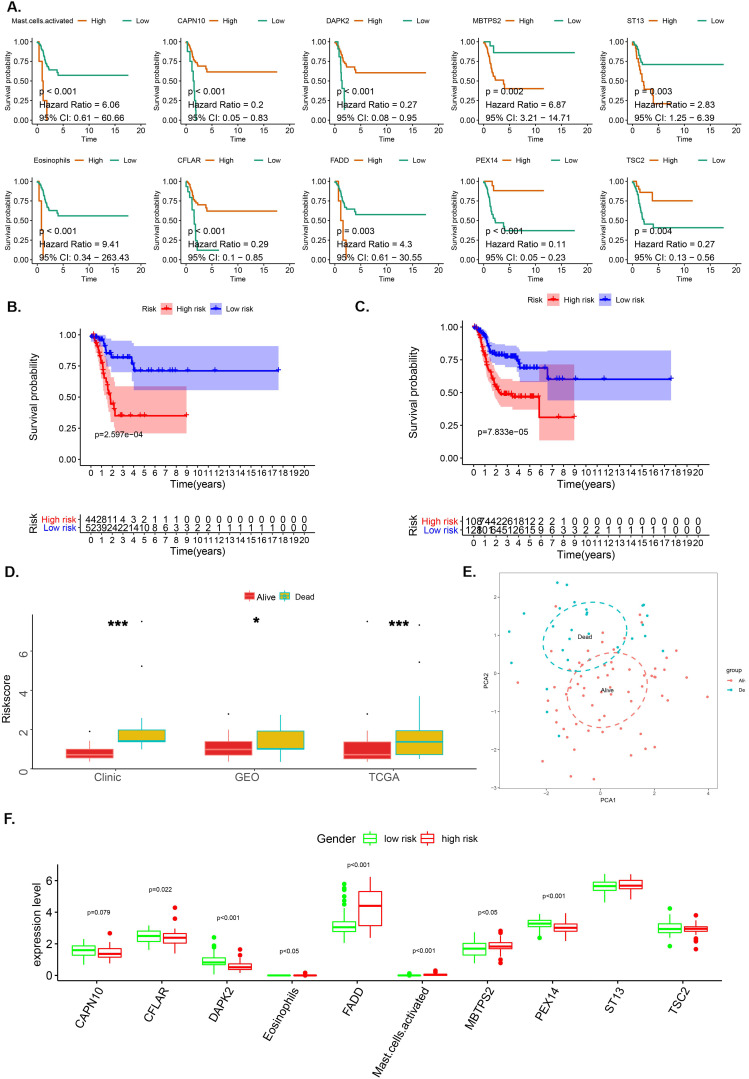
** The OS-associated factors for the prognostic prediction of laryngocarcinoma. A-C.** K-M analyses. K-M curves of OS-associated factors as detected by Lasso Regression analyses (A); The K-M curves of OS survival as per the prognostic risk score (pRS) groups in the TCGA cohort (B); K-M curves of OS survival as per the pRS groups in the entire cohort (C). **D.** Difference levels of pRS in TCGA cohort, GEO cohort and clinical cohort (P < 0.001, K-W test). **E.** Principal component analysis (PCA) reveals that the TCGA laryngeal cancer gene expression data set can be divided into two parts according to pRS. This indicates that pRS has a good prognosis differentiation in laryngeal cancer. The blue dots represent dead samples, while the red dots indicate the survival samples. **F.** Different expression levels between high-risk and low-risk groups.

LASSO analysis aided in the identification of 8 genes (CAPN10, DAPK2, MBTPS2, ST13, CFLAR, FADD, PEX14 and TSC2) and 2 immune cells (Eosinophil and Mast cell) with satisfactory K-M curves performance (Figure 2A) after which we established a prognostic risk scoring model (pRS) using a Cox multivariate regression model. Based on the cut-off value (1.00) drawn from the entire cohort, we classified patients into high pRS group and low pRS group. The Kaplan-Meier curve showed that the risk of death in the high-pIRS group was significantly higher than that in the low-pRS group in TCGA cohort as well as the entire cohort (p <0.001) (Figure 2B-2C). In all patient groups, pIRS as a continuous variable was found to be a powerful independent risk factor for survival (Figure 2D, 2F). According to pRS, the TCGA laryngeal cancer gene expression data set can be divided into two parts. This revealed that pRS can accurately be applied in the prognosis of laryngeal cancer (Figure 2E).

### Overall Survival

**Figure 3:**
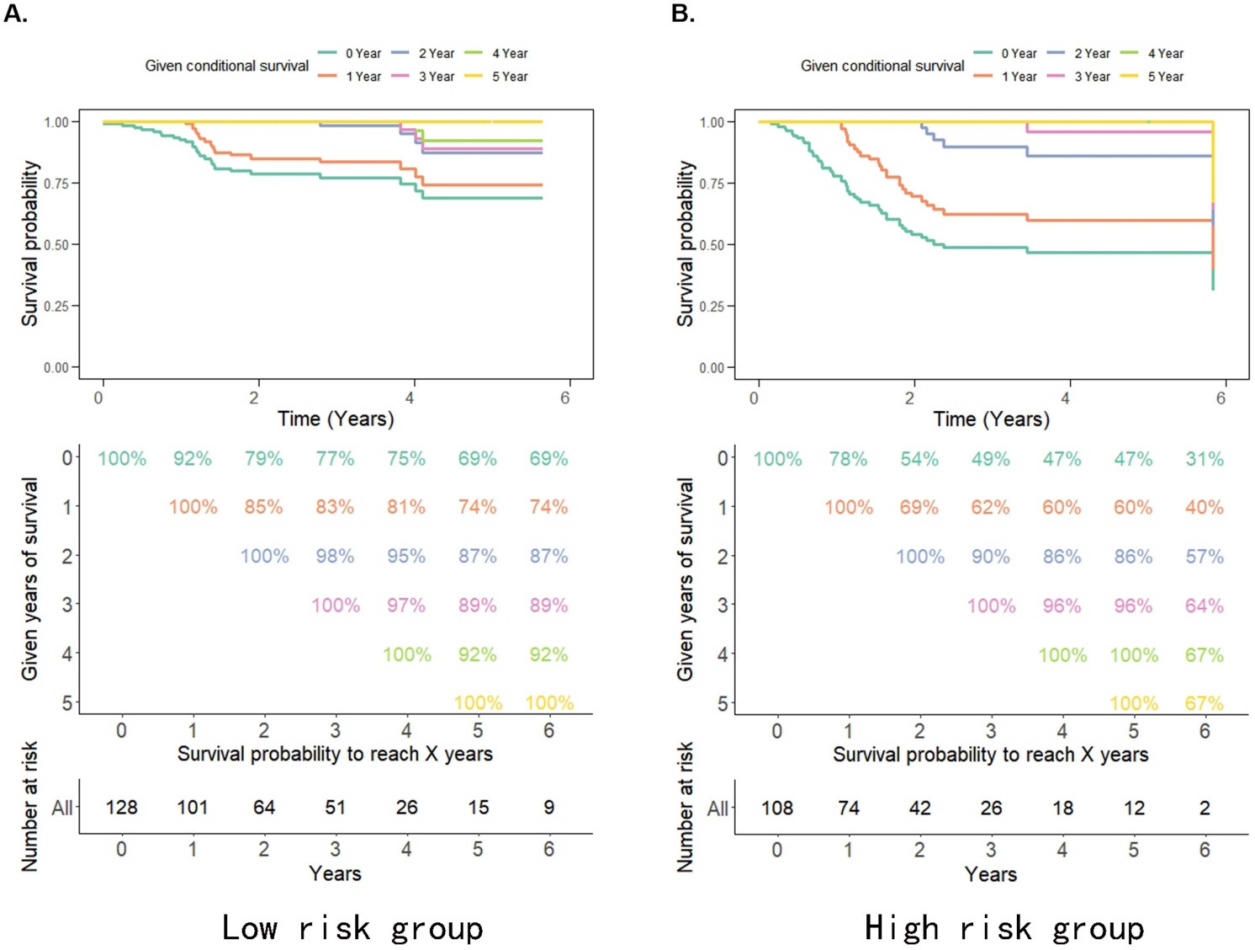
** A-B.** Estimated survival rates in patients given 0-5 years' survival in low/high-risk groups. Each column represents the survival years from surgery and each row represents the percentage of attaining certain total survival time from the survived years point.

The probability of achieving 5-year survival in the low-risk group increased from 69% to 74%, 87%, and 92% per additional year survived (i.e. 1, 2 and 4 years, respectively) better than the probability of achieving 5-year survival in the high-risk group which increased from 31% to 40%, 57%, and 67% per additional year survived (i.e. 1, 2 and 4 years, respectively) (Figure 3A, 3B). Moreover, the survival rate of patients in the high pRS group was lower than that in the low pRS group in the 0-2 years after treatment, whereas the survival rate of the two groups of patients after 3 years of treatment was similar (Figure 3A, 3B). This suggests that the CS rate gradually improved and the survival rate of patients in the high/low-risk groups stabilized after 3 years of treatment. Thus, pRS is vital in predicting the survival rate of patients 0-2 years after treatment and patients with a higher malignant tumor microenvironment.

### Nomogram construction and validation

**Figure 4:**
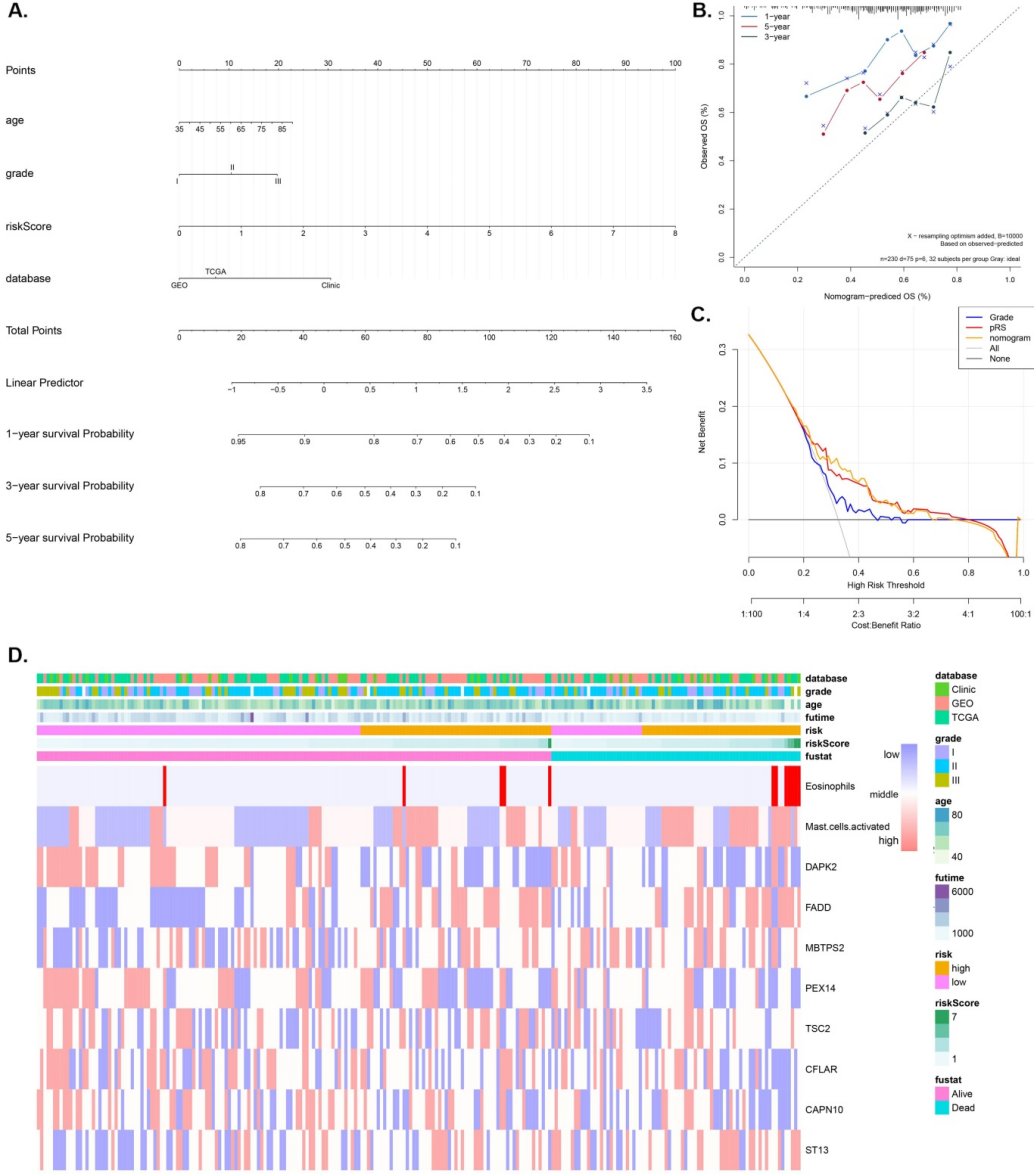
** Nomogram construction and validation. A.** Nomogram for predicting 1-, 3-, and 5-year OS of laryngocarcinoma patients in the entire cohort based on pRS and clinicopathological factors. **B.** A calibration curve of the laryngocarcinoma nomogram. Note: The y-axis is the actual incidence of death whereas the x-axis represents the risk of death. The closer the solid line (the prediction ability of nomogram) matches the dotted line (represents a perfect prediction model), the higher the prediction ability. **C.** Decision curve analyses (DCA) of the prediction model for death-risk in the Grade model, the pRS model, and the nomogram model. **D.** The Heatmap plots of the entire cohort (including the TCGA cohort, GSE27020 cohort, and clinical cohort) for constructing the nomogram.

A nomogram model that integrates the pRS and clinicopathological parameters is shown in Figure 4A. The calibration curves (Figure 4B) show that the prediction nomogram was highly efficient compared to the ideal model. The C-index of this pRS model 0.74 (95% CI, 0.67-0.80) was higher than that of the nomogram model 0.66 (95% CI, 0.58-0.74) and the SEER grade-age model 0.60 (95% CI, 0.58-0.61) (the original data was retrieved from the SEER database, n=9583, Supplementary Table 1) as well as the other grade-age model 0.56 (95% CI, 0.55-0.57) (the original data retrieved from TCGA, GEO and Clinical database), implying that pRS has a better predictive ability in the prognosis of laryngocarcinoma (Table 2). Decision curve analyses (DCA) of the nomogram is shown in Figure 4C, which also revealed that pRS has better predictive ability than the grade-age model. Therefore, the nomogram is suitable for early intervention in predicting the death-risk of laryngocarcinoma. Heatmap plots of the TCGA, GSE27020, and clinical cohorts used in constructing the nomogram are presented in Figure 4B.

### Tumor characteristics analysis of pRS

**Figure 5:**
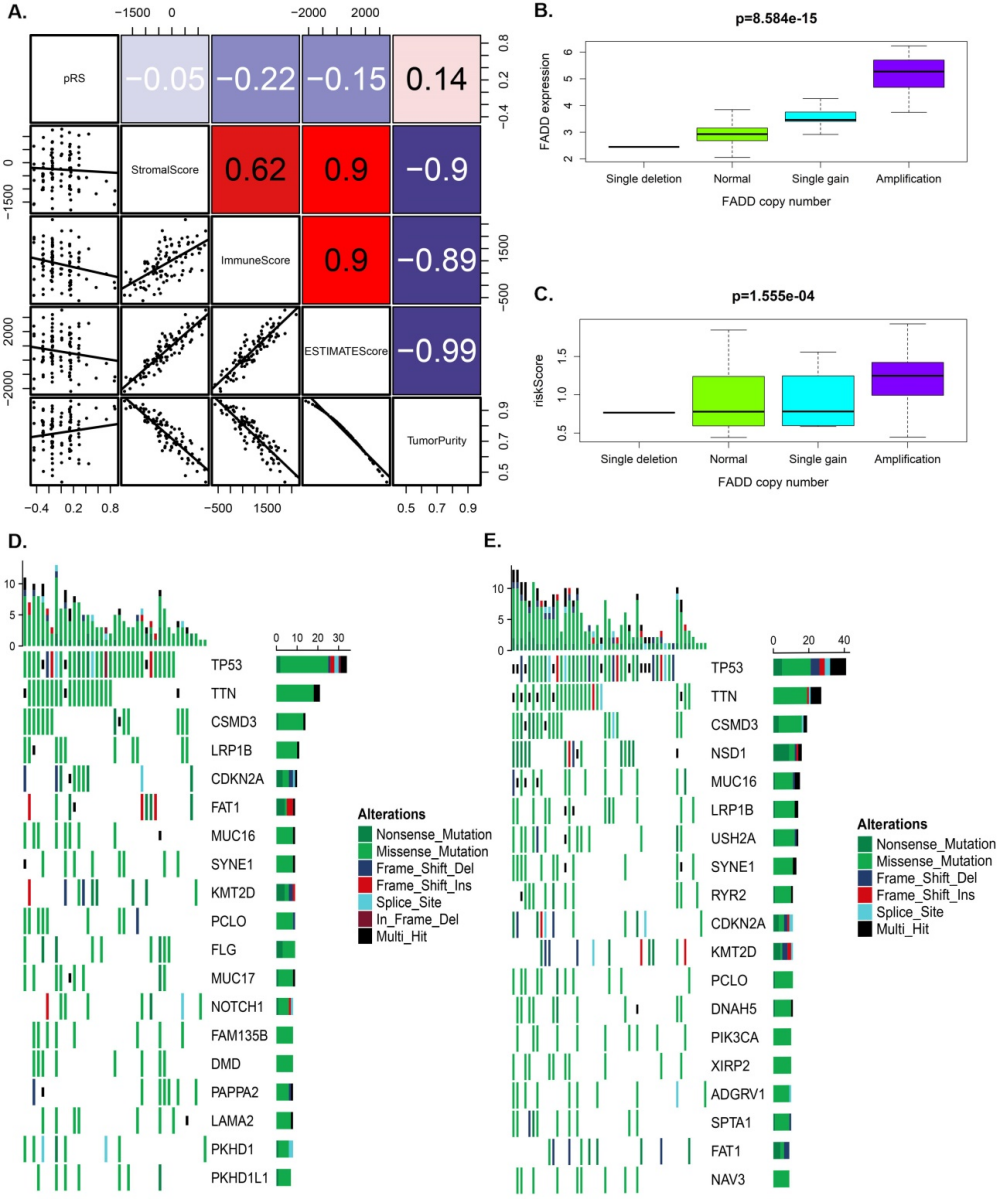
** Tumor characteristics analysis of pRS. A.** A correlations among pRS, StromalScore, ImmuneScore, ESTIMATEScore and TurmorPurity in the TCGA laryngocarcinoma cohort. **B-C.** Differences in FADD expression or pRS between different copy numbers of FADD (p < 0.0001). **D-E.** The waterfall plot of tumor somatic mutation established by genes with high pRS (D) and low pRS (E). The number on the right indicates the mutation frequency in each gene. **F.** Comparison of the pRS with the expression level of PD-1 in laryngocarcinoma. The correlations of laryngocarcinoma from the TCGA cohort are shown.

Figure 5A showed that pRS had A significant correlation with the ImmuneScore, ESTIMATEScore and TurmorPurity (Spearman's correlation, rho=-0.22, -0.15, 0.14, respectively). ssGSEA method was then used to predict the abundance of immune cells in each sample to further study those correlation relationship and a heat map was constructed to visualize the features (Supplementary Figure 1A). It was shown that the pRS score was closely related to a series of microenvironment characteristics (such as TurmorPurity and immune infiltration).

Furthermore, we separately analyzed OS-associated methylated and autophagy associated genes for laryngeal carcinoma with differences in SNP and CNV in the high and the low pRS groups. We found that the pRS-related genes (PEX14, ST13, STK11, TSC2, and CAPN10) exhibited a higher mutation frequency than IKBKB. The location of CNV alteration of OS-associated methylated and autophagy on chromosomes was significantly different between the high pRS group and the low pRS group (Supplementary Figure 1B). Laryngeal cancer patients with high FADD expression exhibited a high mortality rate (Figure 2A). Among these genes, we found that the copy number of FADD was positively correlated with the expression of FADD (Figure 5B). Interestingly, the copy number of FADD was also positively correlated with pRS value (Figure 5C). High pRS group presented less extensive SNP burden than that of the low pRS group (Figure 5D-5E), with the rate of the 10^th^ most significantly mutated genes 18.1% versus 35.8%. These findings suggest that pRS values may be associated with SNP and CNV in laryngeal cancer.

### Analysis between pRS and several important immune checkpoints

**Figure 6:**
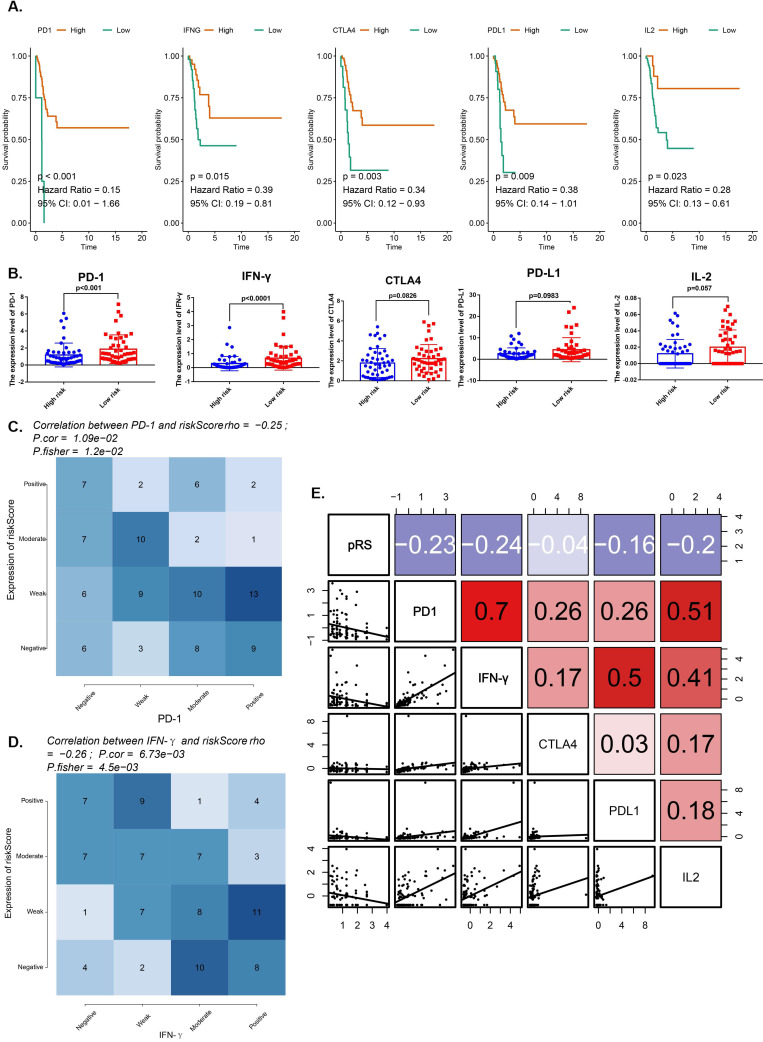
** Analysis for several important immune checkpoints related with pRS. A.** K-M analyses. K-M curves of immune checkpoints (PD-1, IFN-γ, CTLA4, PD-L1, IL-2). **B.** Differences in immune checkpoints (PD-1, IFN-γ, CTLA4, PD-L1, IL-2) expression between high- and low- risk groups. **C-D.** Comparison of expression scores of pRS with those of PD-1 or IFN-γ in laryngeal cancer. The correlations shown are for laryngeal cancer from TCGA cohorts. **E.** Correlations among pRS, PD-1, IFN-γ, CTLA4, PD-L1 and IL-2 in laryngeal cancer (TCGA cohort; n = 96).

We compared the expression of immune checkpoint molecules (programmed cell death 1 (PD-1), cytotoxic T lymphocyte antigen 4 (CTLA-4) and programmed cell death 1 ligand 1 (PD-L1) and several important cytokines (interferon γ (IFN-γ), interleukin 2 (IL-2), transforming growth factor β (TGF-β) in the TCGA-HNSC cohort. All of them with satisfactory K-M curves performance (Figure 6A). PD-1 and IFN-γ expressions were significantly higher in low risk group (Figure 6B). We reported a significant negative correlation of pRS with PD-1 and IFN-γ in the TCGA cohort (Figure 6C,D). Furthermore, patients with better prognosis showed significantly high expression of PD-1 (Figure 5G), suggesting the potential effect of anti-PD-1 immunotherapy. We also rank those pRS genes (TSC2, DAPK2, CFLAR, CAPN10, PEX14, ST13, FADD and MBTPS2) based on dysfunction and risk score by using TIDE (Supplementary Figure 1). All of them were enriched in the most of those researches (E-MTAB-179, GSE12417_GPL570, ICD_Gide2019_PD1+CTLA4, ICB_Riaz2017_PD1 Ipi_Naive, etc.) (Supplementary Figure 1). Notably, our work shows that pRS is significantly correlated with PD-1 and IFN-γ immunophenotype, thus establishing pRS may help predict the effectiveness of anti-PD-1 immunotherapy.

### GSVA and GSEA enrichment of the pRS subtypes

**Figure 7:**
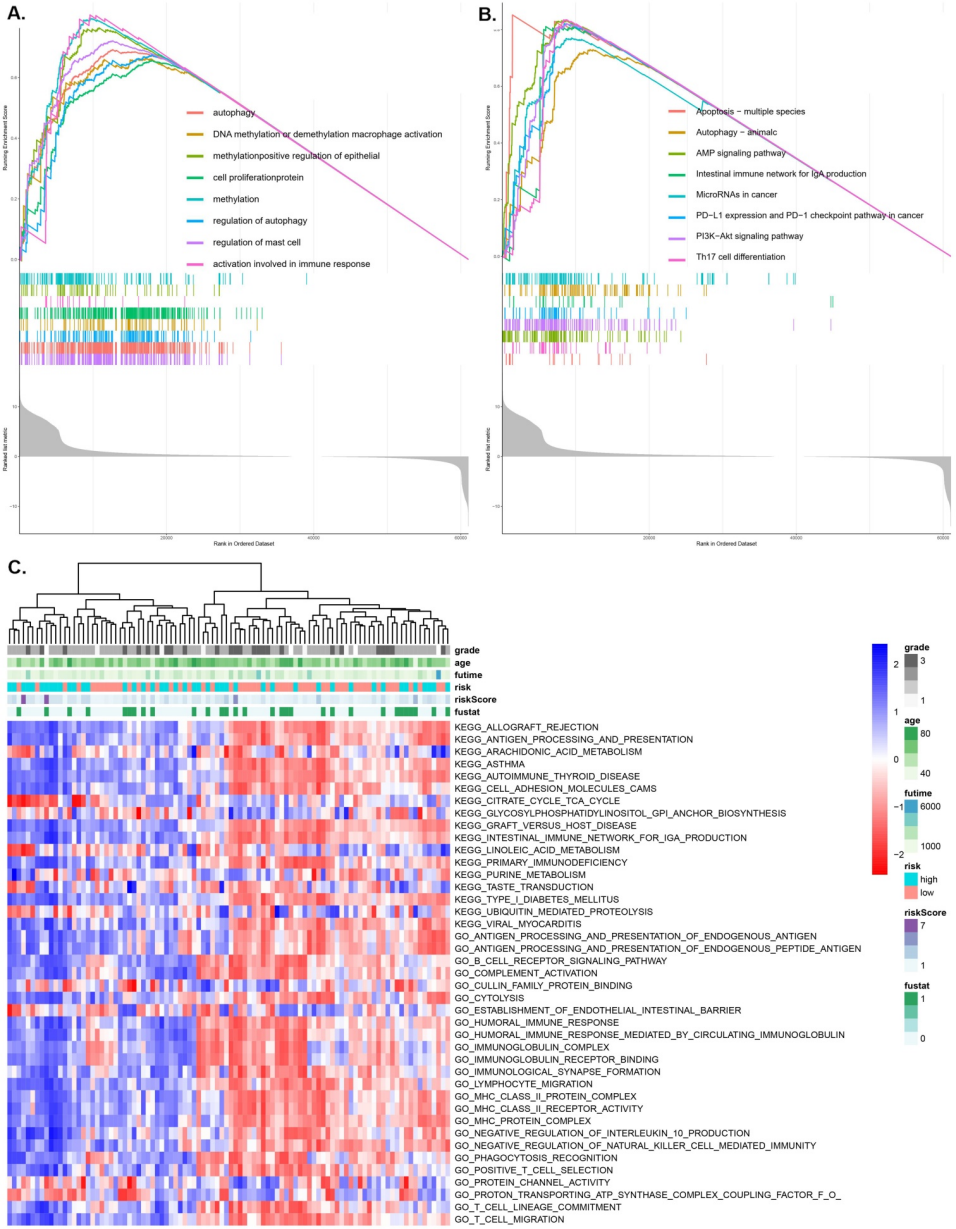
** GSEA (A and B) and GSVA (C) analysis displaying the biological processes and pathways associated with pRS. There is a significant correlation between the low and high pRS groups. A.** GO function enrichment analysis. **B.** KEGG enrichment analysis. **C.** The heatmap was used to visualize the KEGG pathways and GO function in laryngocarcinoma between high- and low-pRS groups (p<0.05).

Using the GO function enrichment analysis of GSEA, we revealed pRS-related functions, such as autophagy, DNA methylation or demethylation, macrophage activation, methylation, positive regulation of epithelial cell proliferation, protein methylation, regulation of autophagy and regulation of mast cell activation (Figure 7A). KEGG pathways enrichment analysis of pRS-related pathways revealed the apoptosis-multiple species, autophagy, AMP signaling pathway, intestinal immune network for lgA production, microRNAs in cancer, PD-L1 expression and PD-1 checkpoint pathway, PI3K-Akt signaling pathway and Th17 cell differentiation (Figure 7B). Additionally, we revealed a high correlation between pRS and PD-L1 expression as well as PD-1 checkpoint pathway, an indication that PD1-related immunotherapy may potentially exert positive benefits on laryngeal cancer patients.

As we can see in the heatmap,there are remarkable differences between high- and low-pRS groups in KEGG pathways and GO function patterns, which are quantified by GSVA analysis (Figure 7C). It is worth noting that pRS was significantly correlated with various immune-related processes including primary immunodeficiency, humoral immune response, immunoglobulin receptor, MHC class II protein complex, lymphocyte migration, T cell migration and so on. Based on these findings, it is evident that the evaluation of pRS can reflect immune cell infiltration in laryngeal cancer, as well as the functions of autophagy and methylation-related pathways.

### Predicting pRS-related ceRNA network

“LIMMA”R package was also used to identify laryngocarcinoma-related circRNAs between laryngeal cancer and normal samples, which were showed in Supplementary Figure 2A. Based on the miRTarBase and miRWalk databases, linkages between pRS-related mRNAs and pRS-related miRNAs are demonstrated in Supplementary Figure 2B. In total, 10 pRS-related mRNAs (COX6B2, EGFR, CD274, LMOD2, CAV1, MB, CREG2, PHYHIP, TNNI1, and SPRR2B) were likely influenced by the down-regulation of hsa-miR-552. Besides, 3 pRS-related mRNAs (PNMAL1, UBD and ADAMTS15) were modulated by hsa-miR-3923. hsa-miR-548k targeted 15 pRS-related mRNAs (OLR1, IL33, TGFBI, F12A1, SPIB, WFDC12, PRSS23, MICALCL, SMPX, NOTUM, PPBP, HAS2, KRT38, DEFA6 and TUBB2B). Other pRS-related mRNAs were proposed to be potentially regulated by hsa-miR-599 and hsa-miR-592, respectively.

**Figure 8:**
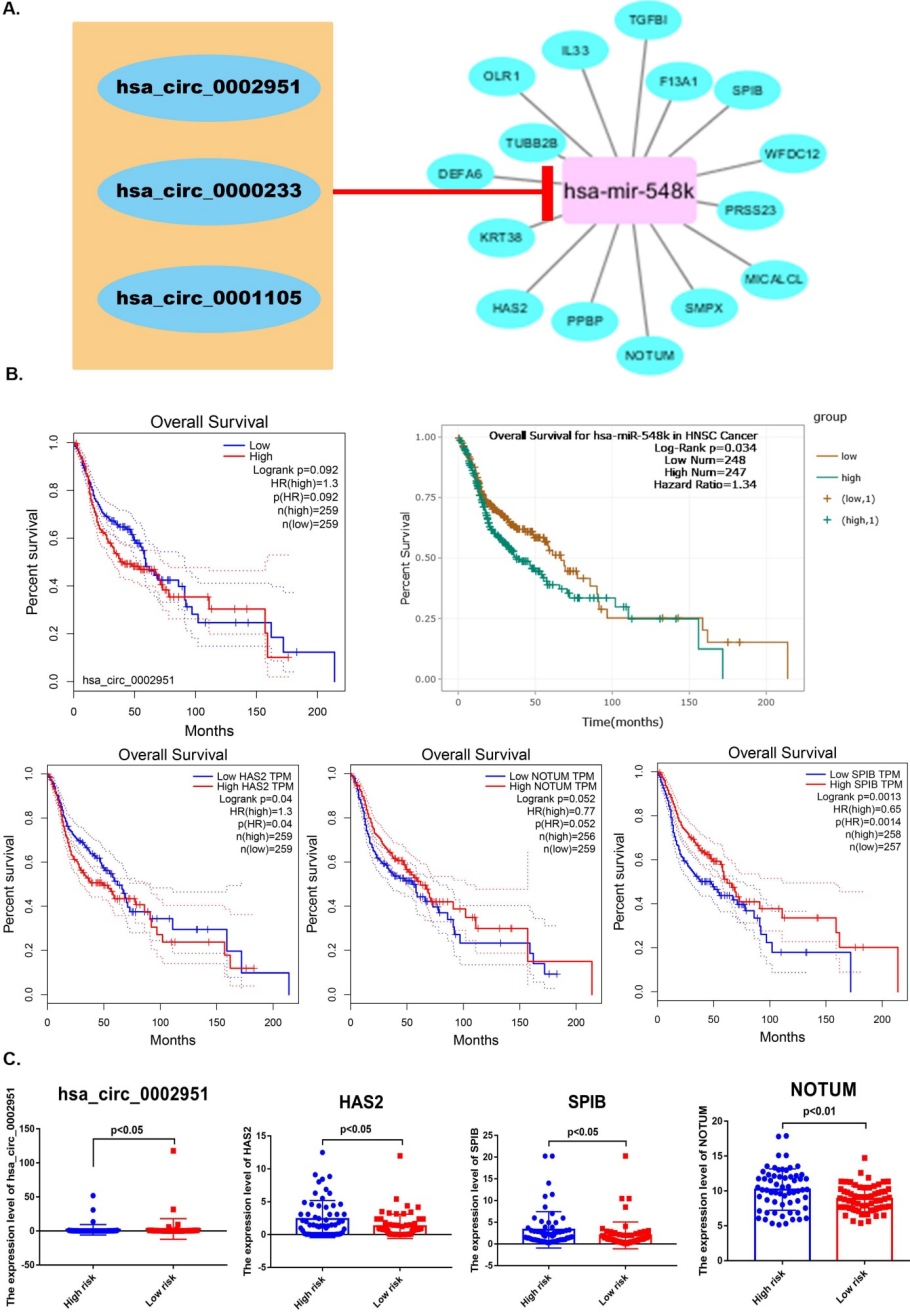
** A.** A ceRNA network is constructed. The regulatory network between laryngocarcinoma-related circRNAs, pRS-related mRNAs and pRS-related miRNAs. **B.** K-M curves of genes from the ceRNA network (hsa_circ_0002951, hsa-miR-548k, HAS2, NOTUM and SPIB). **C.** Differences in genes (hsa_circ_0002951, HAS2, NOTUM and SPIB) expression between high- and low- risk groups.

After that, Circular RNA Interactome was used to reveal correlation between laryngocarcinoma-related circRNAs and pRS-related miRNAs. We predicted that miR-548k might target these three cicRNAs (hsa_circ_0002951, hsa_circ_0000233 and hsa_circ_0001105). Based on this the regulatory network between laryngocarcinoma-related circRNAs, pRS-related mRNAs and pRS-related miRNAs was constructed (Figure 8A). Among those genes, hsa_circ_0002951, hsa-miR-548k, HAS2, NOTUM and SPIB were found to be with satisfactory K-M curves performance (Figure 8B). HAS2, hsa_circ_0002951, NOTUM and SPIB expressions were significantly higher in low risk group (Figure 8C). Those results suggesting a potential positive correlation between HAS2, hsa_circ_0002951, NOTUM and SPIB.

**Figure 9:**
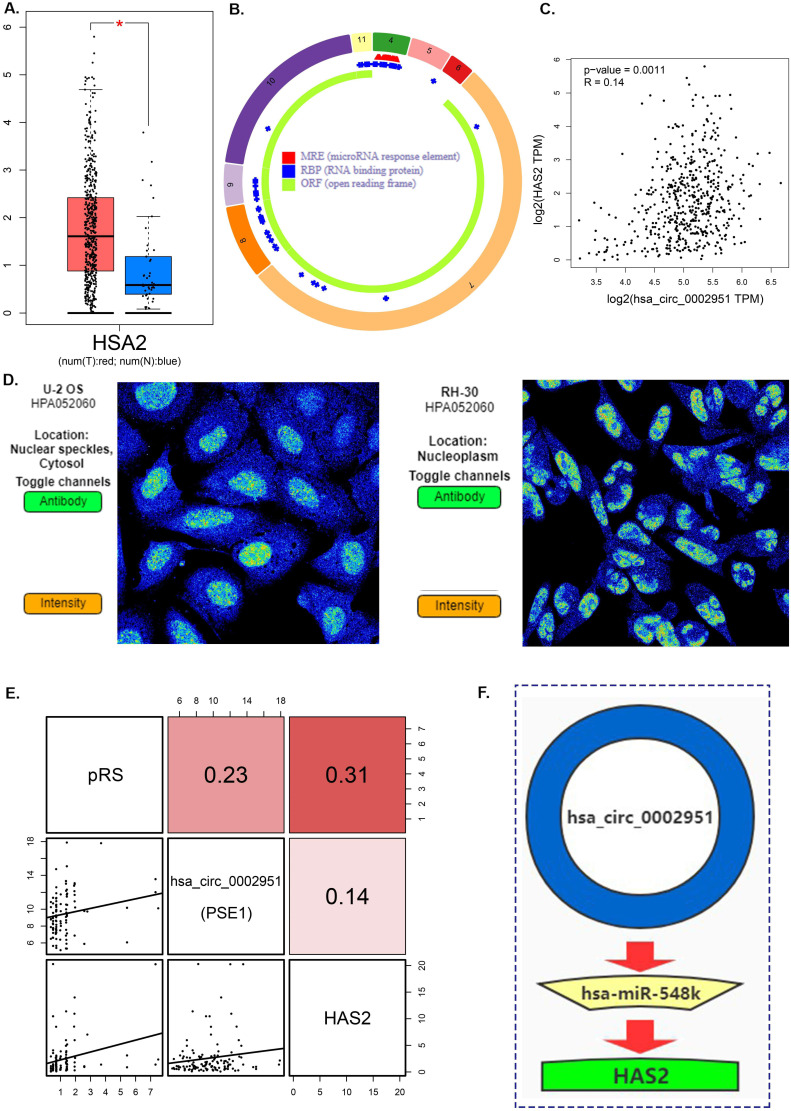
** A.** Differences in HAS2 expression between tumor samples and normal samples. **B.** hsa_circ_0002951 structures were obtained from the circRNA website. Yellow represents the open reading frame, red represents the miRNA bind position and blue represents the position where the protein may bind. **C.** Correlations among HAS2 and hsa_circ_0002951 expression in laryngeal cancer. **D.** The immunofluorescent analysis of HAS2 is carried out in two different cell lines (U-2 OS and RH-30). And HAS2 was found mainly localized to the nuclear speckles. **E.** Correlations among pRS, HAS2 and hsa_circ_0002951 in laryngeal cancer (TCGA cohort; n = 96). **F.** A model showing the inhibitory effect of circ_0002951/miR-548k/HAS2 pathway in laryngeal cancer was established. hsa_circ_0002951 as a miR-548k sponge regulates HAS2 expression and affects the survival rate of patients with laryngeal cancer.

Hyaluronan Synthase 2 (HAS2) is a key ubiquitous enzyme located at the plasma membrane that synthesizes hyaluronan and extrudes these long polysaccharides into the extracellular space. We found that HAS2 was highly expressed in tumor samples (Figure 9A). The basic structural pattern of hsa_circ_0002951 is shown in Figure 9B. Besides, the immunofluorescent analysis of HAS2 was carried out in two different cell lines (U-2 OS and RH-30) (Figure 9D). And HAS2 was found mainly localized to the nuclear speckles, which suggests HAS2 may be involved in tumor proliferation. As previous researches indicate, HAS2 related pathway represents promising novel anti-cancer therapy targets 70-73, which is similar to our conclusion (Supplementary Figure 3). Studies have shown that 4-MU inhibits HAS2 not only by decreasing the levels of the enzymes involved with its synthesis but also by sequestering glucuronic acid, ultimately inhibiting proliferation, invasion, and migration in prostate, breast, ovarian and melanoma carcinomas 74. Further analysis indicated that hsa_circ_0002951 and HAS2 have a strong positive correlation with p-value<0.0011 (Figure 9C). In the TCGA cohort, correlation analysis showed a significant positive correlation among pRS, hsa_circ_0002951 and HAS2 (Figure 9E). Therefore, a model showing the inhibitory effect of circ_0002951/miR-548k/HAS2 pathway in laryngeal cancer was established (Figure 9F). We suggested that hsa_circ_0002951 as a miR-548k sponge may regulate HAS2 expression and affect the survival rate of patients with laryngeal cancer. In conclusion, the identified pRS-related ceRNA network provides potential therapeutic targets and biomarkers of laryngocarcinoma.

### Single‑cell RNA‑seq analysis reveals high cell heterogeneity

**Figure 10:**
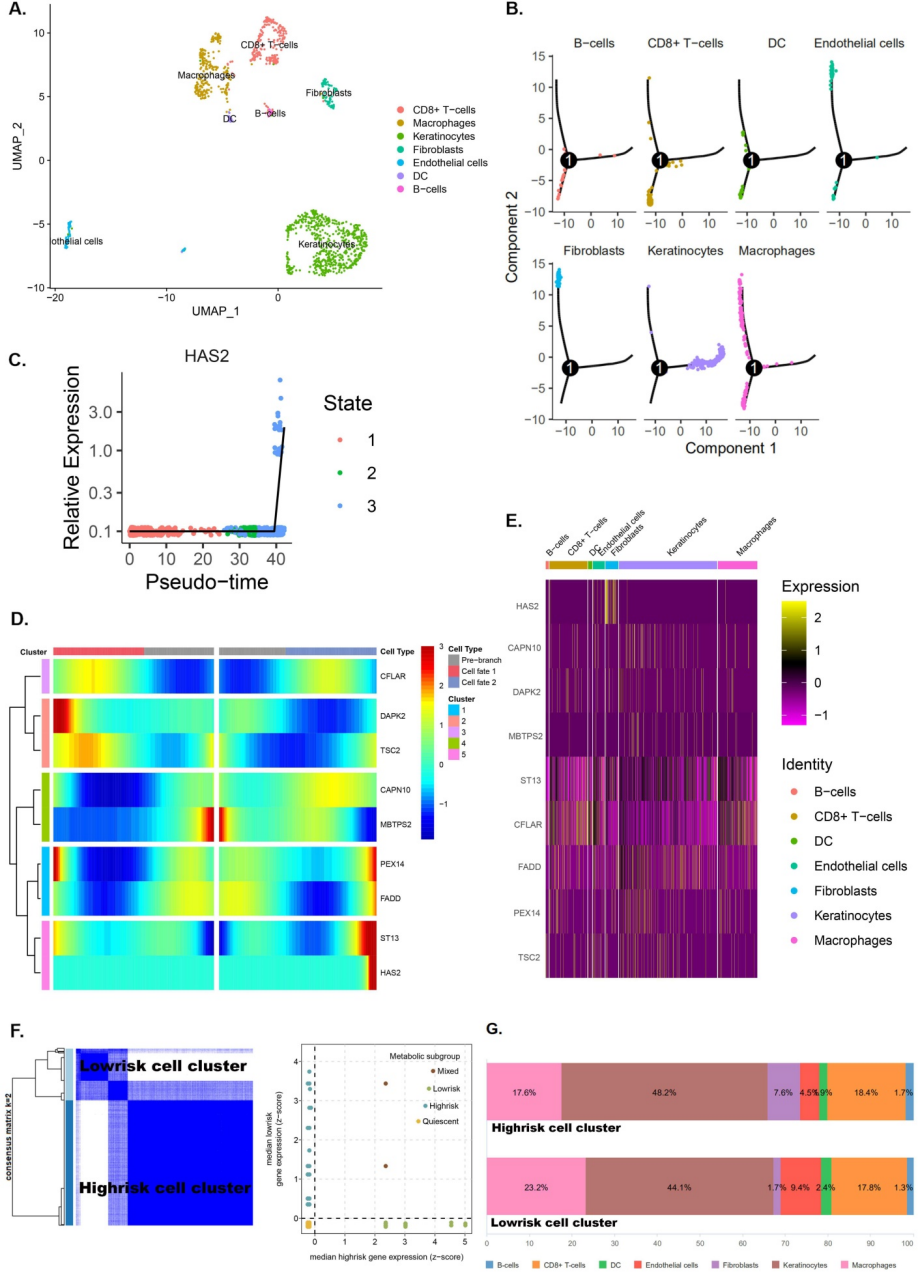
** Single‑cell RNA‑seq analysis reveals high cell heterogeneity. A.** Cells from laryngeal cancer were all annotated by CellMarker and singleR. **B.** Trajectory analysis revealed laryngocarcinoma cells with distinct differentiation patterns. **C-D.** Trajectory analysis revealed the differential expression of pRS-related genes (CFLAR, DAPK2, TSC2, CAPN10, MBTPS2, PEX14, FADD, ST13 and HAS2) at different pseudo-time. **E.** 8 pRS-related genes (CFLAR, DAPK2, TSC2, CAPN10, MBTPS2, PEX14, FADD and ST13) clustering analysis divided laryngocarcinoma cells into two clusters (the low risk cell cluster and the high risk cell cluster) with a well discriminatory power. **F.** A bar chart of cell classification percentage in the low risk cell cluster and the high risk cell cluster.

Seurat package in R3.6.3 was used for quality control and the remaining 1229 cells were normalized by Seurat package and PCA was completed for preliminary dimension reduction (Supplementary Figure 4A, C, D). ANOVA revealed 2000 corresponding marker genes in all laryngeal cancer cells and labeled the top 20 marker genes in each cell cluster (Supplementary Figure 4B, E). According to the expression patterns of the marker genes (Figure 10A), annotation of the clustering results of the UMAP method downscaled with singleR and CellMarker.

Trajectory analysis shows significant differentiation cell heterogeneity in laryngeal cancer tissues (Supplementary figure 4F and Figure 10B). In laryngeal cancer tissues, fibroblasts and endothelial cells have a much later pseudotime, while keratinocytes have a much earlier pseudotime (Figure 10B). Moreover, trajectory analysis also demonstrates that the expression of genes (CFLAR, DAPK2, TSC2, CAPN10, MBTPS2, PEX14, FADD, ST13 and HAS2) change with pseudo-time (Figure 10 C,D). The expression of pRS-related genes (CFLAR, DAPK2, TSC2, CAPN10, MBTPS2, PEX14, FADD, ST13 and HAS2) in different cells is shown in Figure 10E.

Clustering analysis of eight pRS-related genes (CFLAR, DAPK2, TSC2, CAPN10, MBTPS2, PEX14, FADD and ST13) divided laryngocarcinoma cells into low and high risk cell clusters (Figure 10F). Interestingly, fibroblasts were found mainly located in laryngocarcinoma cells (Figure 10G). This suggests that a high degree of fibroblasts may be responsible for the poor prognosis of high pRS in laryngocarcinoma.

### Fibroblasts-related intercellular interactions and ligand-receptors analysis

**Figure 11:**
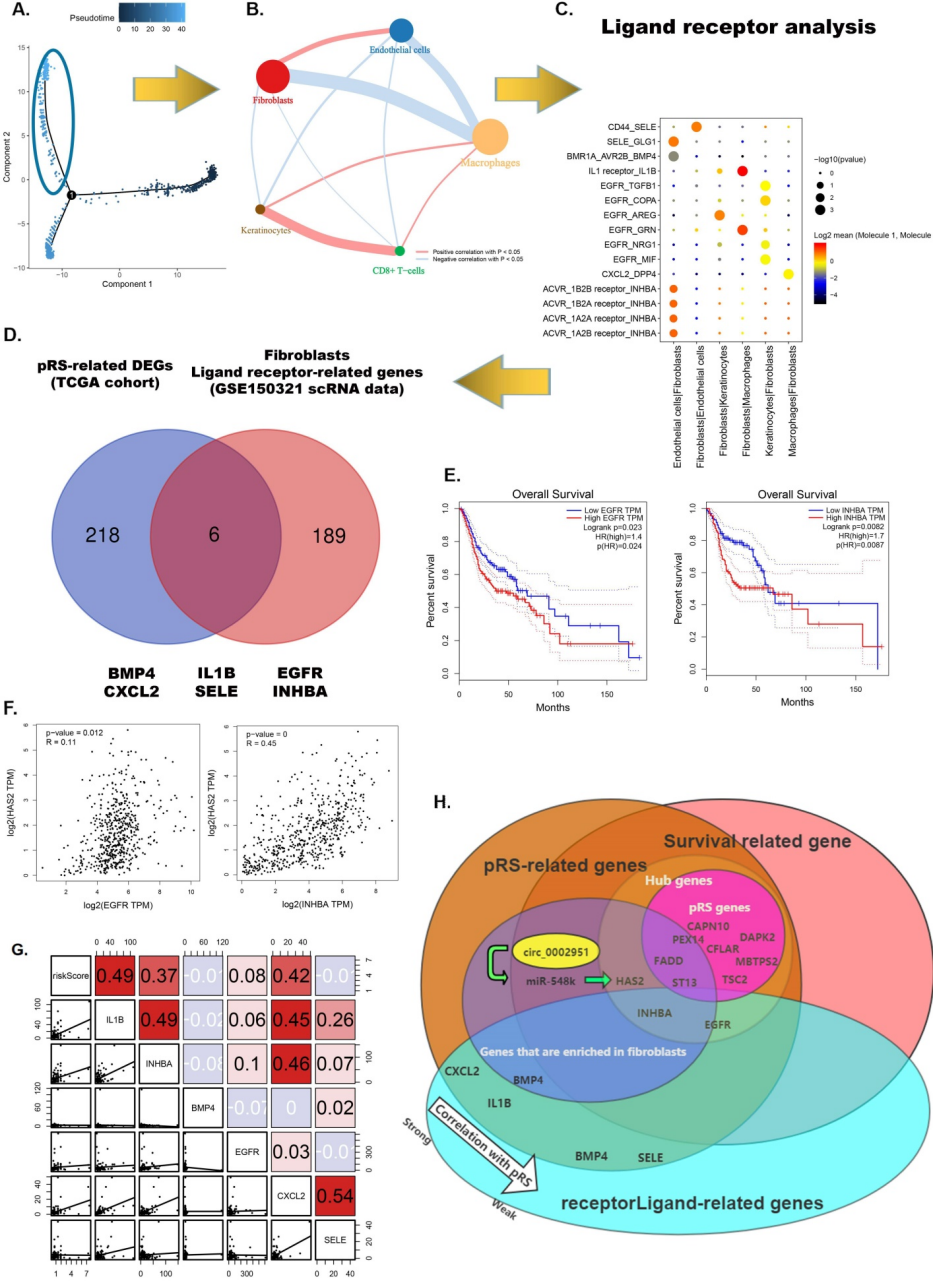
** Fibroblasts related intercellular interactions and ligand-receptors analysis. A.** Trajectory analysis revealed laryngocarcinoma cells with distinct differentiation patterns. **B.** Correlation analysis of intercellular interactions in regions with a pseudotime greater than 35. **C.** The receptor-ligand interaction within fibroblasts. **D.** Veen diagram showed intersection of genes between pRS-related DEGs (TCGA cohort) and ligand receptor-related genes in fibroblasts. **E.** Survival analysis of EGFR and INHBA based on TCGA laryngeal cancer samples. **F.** Correlation analysis of HAS2 with EGFR and INHBA respectively based on TCGA database. **G.** Correlations among pRS, IL-1B, INHBA, BMP4, EGFR, CXCL2 and SELE in the TCGA cohort. **H.** Diagram of the relationship between genes in this paper.

From the previous analysis, we found that fibroblasts may be associated with high pRS scores and fibroblasts were predominantly distributed in regions with a pseudotime greater than 35 (Figure 11A). We performed a correlation analysis of cell-to-cell interactions for cells in this region (Figure 11B). Fibroblasts were found to have a significant positive correlation with endothelial cells and a significant negative correlation with macrophages. This suggests that macrophages increase first as the tumor progresses malignantly, whereas infiltration of fibroblasts and endothelial cells may signify failure of the body's immune defenses and the occurrence of malignant tumor progression. To further examine the differences and commonalities in information exchange between cells, we used CellPhoneDB to infer cell-to-cell communication. Figure 11C shows the most critical receptor-ligand interactions in the fibroblasts. A Venn diagram shows the intersection of six genes (BMP4, CXCL2, IL-1B, SELE, EGFR and INHBA) in pRS-related DEGs (TCGA cohort) and ligand receptor-related genes in the fibroblasts. Among them, EGFR and INHBA were found to be significantly correlated with laryngeal cancer patient prognosis, HSA2 expression and pRS score (Figure 11 E-G and Supplementary Figure 5). This suggests that EGFR and INHBA can serve as prognostic hub genes for laryngeal cancer collectively with pRS genes (CFLAR, DAPK2, TSC2, CAPN10, MBTPS2, PEX14, FADD and ST13). A more visual representation of the interrelationship diagram of these genes can be found in Figure 11H.

### Hub pRS-related gene signatures predict laryngocarcinoma cell types

**Figure 12:**
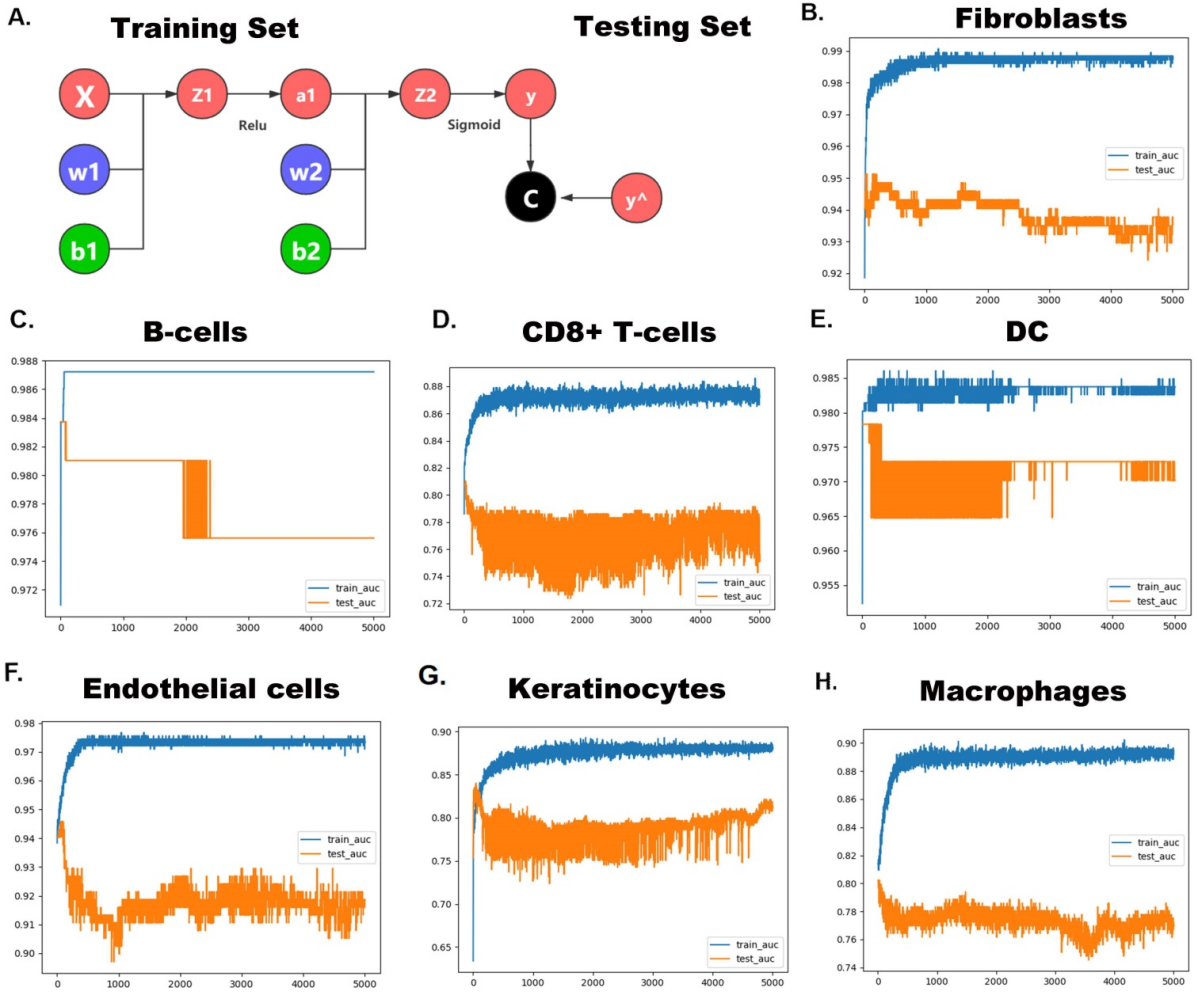
** Hub pRS-related genes signature predict laryngocarcinoma cell types. A.** A Schematic diagram of the neural network. **B-H.** The ROC plot in the traning set and the validation set validated the accuracy of those network's prediction capacity.

Since these pRS-related hub genes (EGFR, INHBA, CFLAR, DAPK2, TSC2, CAPN10, MBTPS2, PEX14, FADD and ST13) show strong heterogeneity in different cells, we speculate that these genes alone can predict cellular composition to reflect the microenvironment of tumor tissues. Therefore, we developed a neural network-based model to predict cell types in laryngeal cancer tissues using these hub genes. The construction of the neural network is shown in Figure 12A. The area under the curve (AUC) of ROCs performed well (Figure 12B-H). This suggests that this model has good predictive power, especially in predicting fibroblasts (AUC ≈ 0.99), DC (AUC ≈ 0.985) and endothelial cells (AUC ≈ 0.975). This signifies that these genes have great potential to map the tumor microenvironment.

## Discussion

The comprehensive application of methylation/autophagy-related genes (MARGs) and immune cells as specific markers for the analysis of laryngeal cancer microenvironment has not been previously described. Here, 8 MARGs and 2 immune cells were selected for constructing the prognostic risk scoring system (pRS). It could provide intelligent advice for clinicians legitimately tailor the treatment plan and help researchers understand the microenvironment of laryngocarcinoma using simple laboratory detection, which could greatly promote individualized therapy and provides immunotherapy strategies.

A systematic analysis of the relationship between immune/methylation/autophagy signatures, laryngeal carcinoma prognosis and the tumor microenvironment was performed in this study. The combination of single-cell and transcriptome laryngeal carcinoma landscape analyses explores the link between the tumor microenvironmental and prognostic characteristics.

In brief, 8 MARGs (CAPN10, DAPK2, MBTPS2, ST13, CFLAR, FADD, PEX14 and TSC2) and 2 immune cells (Eosinophil and Mast cell) were obtained to establish the pRS scoring system. The pRS was used to establish a risk prediction model for the prognosis of laryngeal cancer. Furthermore, pRS was found highly associated with SNP, CNV, ICB therapy and the tumor microenvironment. The circ_0002951/miR-548k/HAS2 pathway was also brought up, which indicates potential therapeutic targets and biomarkers of laryngocarcinoma. Single cells were then genotyped and fibroblasts were found enriched in high risk cell clusters at the scRNA-seq level. Fibroblast-related ligand-receptor interactions were revealed to define hub genes. A neural network-based deep learning model based on these pRS-related hub gene signatures was also established with a high accuracy in cell type prediction.

Our study screened out molecular markers associated with the prognosis of laryngeal cancer and established a risk scoring system (pRS) to predict its prognosis. Relying on transcriptomics data to describe the tumor microenvironment computationally is a promising approach that overcomes the technical limitations of IHC, and can further characterize diverse immune populations with multiple functional phenotypes in a large patient cohort much more readily than with IHC. In this work, we applied the newly developed algorithm “CIBERSORT” in establishing a prognostic risk score (pRS) based on 8 m^6^A-RNA methylation/autophagy-related genes (CAPN10, DAPK2, MBTPS2, ST13, CFLAR, FADD, PEX14 and TSC2) and 2 immune cells (Eosinophil and Mast cell). The excellent predictive ability of pRS for laryngeal cancer was further confirmed through subsequent C-index analysis and using the ROC curve. However, according to the guidelines established by Altman et al. 75, only signatures validated in independent cohorts of patients with full clinical annotation available could be applied clinically. Therefore, the prognostic value of pRS model is to be validated. Since the current high-throughput gene expression measurement technology has been well developed, we believe that our pRS classifier has a higher potential to be translated into clinical practice.

Moreover, based on our findings, we revealed that the immune system and cellular autophagy process influence the prognosis of laryngeal cancer. The prognostic model of pRS can stratify laryngeal cancer patients and effectively identify patients with a high risk of death, thereby promoting individualized treatment based on patient risk and revealing possible new targets for laryngeal cancer treatment. In addition, we found that in 0-2 years after treatment, the survival rate of patients in the high pRS group was lower than that in the low pRS group, and there were almost indistinguishable survival rates between the two groups after 3 years of treatment. This suggests that the CS rate gradually improves over time, and the survival rate of patients in both groups will gradually stabilize. Most importantly, since patients prefer clear survival information, the intuitive display of this information helps them cope with the fear of relapse or death, and pave the way for personalized follow-up plans 76-78.

In this study, a sharp link between immune cell infiltration, the expression of autophagy genes and m^6^A methylation related genes were reported. Notably, 8 autophagy-related genes (CAPN10, DAPK2, MBTPS2, ST13, CFLAR, FADD, PEX14 and TSC2) and 2 immune cells (Mast cells and Eosinophils) were revealed to be highly associated with tumor prognosis. Furthermore, a comprehensive analysis showed that the pRS composed of the above 8 genes and 2 immune cells can independently predict the prognosis of laryngeal carcinoma. A higher pRS score of patients with laryngeal cancer implied worse prognosis. We noted that pRS scores were significantly associated with differences in mRNA transcriptome, immune infiltration, autophagy, and methylation biological pathways. Therefore, a comprehensive assessment of the state of autophagy, methylation and immune infiltration will improve the understanding of the tumor microenvironment of laryngeal cancer cells 10.

In addition, our work showed a significant negative correlation between pRS and tumor mutation burden, and a positive correlation between pRS and DNA copy number variation 10. Consistent with previous studies, genetic aberrations, such as DNA copy number variation (CNV) and single nucleotide mutation (SNP), have strong links with the occurrence and prognosis of head and neck squamous cell carcinoma 79.

PD-1 expression is negatively correlated with pRS. Therefore, patients with low PD-1 expression have a worse prognosis. This reveals that PD-1 may play a strong part in the prognosis of laryngeal cancer patients, consistent with previous researches 80. We also found that pRS scores are significantly associated with numerous immune checkpoint markers, inflammatory factors, and immune activation pathways. Thus, the immunotherapy effect of the patient group with high pRS may be advanced. However, there is an urgent need to explore the potential value of pRS scores in predicting the response of laryngeal cancer patients to immunotherapy.

Researchers have explored the importance of immune cell infiltration in predicting the prognosis of various solid tumor types 81-83. In our survival analysis, we found that Mast cell activation and eosinophils are associated with poor prognosis 1. To our knowledge, eosinophils have been implicated in angiogenesis and tumor metastasis 84, and elevated levels of eosinophils suggest poor prognosis for cancer patients, which is consistent with previous findings 85. Of note, mast cells can participate in the formation of blood vessels and lymphatic vessels 86, 87 as well as the occurrence and progression of tumors 88-90. Previous studies have also found that mast cells can promote the proliferation of thyroid cancer cells 91, and may play an important role in the process of Epithelial-to-mesenchymal (EMT) 92. Our research further validated these findings.

Most genes constituting the pRS component are closely related to the prognosis of various malignant tumors 93. Consistent with previous studies, FADD gene copy number variation and protein expression are potential prognostic markers for squamous cell carcinoma of the head and neck. Notably, patients with FADD copy number amplification and high protein expression have the shortest disease-free survival 93. Down-regulation of the TSC2 gene can result in the accumulation of Rheb-GTP, consequently activating classic mTORC1 and promotes the growth and proliferation of cells, which causes tumors 94, 95. In tumors such as B-lymphoma and myeloma, silencing of DAPK2 methylation is associated with poor tumor prognosis 96. In neuroblastoma, PEX14 down-regulation was found to be associated with tumor progression and poor prognosis 97. In a study by Nader Shakibazad et al., they found that IFAP syndrome caused by the MBTPS2 gene may be a risk factor for malignant tumors 98. However, the role of some genes still needs further exploration, for instance, the role of ST13 and CAPN10 in cancer development should be elucidated 99, 100. Another study by Simone Fulda revealed that CFLAR participates in the regulation of various cell death signaling pathways such as apoptosis, necrosis, and autophagic cell death. Besides, CFLAR abnormal expression is related to the prognosis of human cancer. Thus, targeted therapy CFLAR may offer a feasible anti-cancer treatment strategy.

The study of tumor cell heterogeneity allows for better understandings of tumor progression 101. Laryngeal cancer single-cell data was clustered by pRS genes and fibroblasts associated with poor prognosis were postulated in this research. Malignant proliferating cells and fibroblasts share the tumor margin in laryngeal cancer 101. Numerous previous studies have confirmed that fibroblasts are one of the major components of TME and can establish dangerous associations with other TME cells, thereby creating a tumor-supporting environment in laryngeal cancer 102. Our research suggests that fibroblasts have a significant positive correlation with endothelial cells. As result, CellPhoneDB was used to infer the cell-cell communication of each subtype by receptor-ligand interaction. Our study suggests that EGFR and INHBA from fibroblasts may lead to malignant transformation of laryngeal cancer. Survival analysis suggests that high expression of EGFR and INHBA is significantly associated with poor prognosis for laryngeal cancer 2. Additionally, INHBA-AS1 also shows to be a possible target and prognostic marker for the treatment of multiple cancers 103-105. Furthermore, a neural network-based deep learning model was also established to predict laryngocarcinoma cell types using pRS-related hub gene signatures. Remarkably, the pRS-related hub genes-based neural network showed high accuracy in the training set as well as the testing set. Therefore, those results highlighted the importance of pRS-related hub genes for exploring the tumor microenvironment.

In this work, we revealed that the pRS score can be used to comprehensively evaluate the prognosis of patients with laryngeal cancer and guide on more effective treatment strategies in clinical practice. Also, pRS can be used to assess the tumor microenvironment, DNA copy number variation, and tumor mutation burden in patients with laryngeal cancer. The ceRNA network and the hsa_circ_0002951/hsa-miR-548k/HAS2 pathway constructed based on pRS may help in revealing the tumor lethal mechanism. Of note, a high correlation between pRS and PD-1 expression may provide insights into devising new drug combination strategies or new immunotherapy drugs. In particular, fibroblasts were found enriched in high pRS cluster and more hub genes (EGFR and INHBA) were then defined by ligand-receptor analysis. Those pRS-related hub genes (EGFR, INHBA, CFLAR, DAPK2, TSC2, CAPN10, MBTPS2, PEX14, FADD and ST13) were found can predict cellular composition to reflect the microenvironment of tumor tissues with high accuracy. Our findings, therefore, provide new ideas for identifying high-risk laryngeal cancer patients, improving their clinical response to immunotherapy and promoting future personalized cancer treatments.

However, this research inevitably has some limitations. First, the amount of data published in the public data set is limited, thus the clinical and pathological parameters analyzed are not comprehensive, which may lead to potential errors or biases. Second, all data series downloaded to construct the pRS model were retrieved from the TCGA and GSE27020 data sets. Therefore, caution should be exercised when applying the conclusions of this study to patients in Asian countries. However, pRS can be optimized in the future to make it more suitable for clinical use. Third, the prognostic model still needs further validation in other independent queues. Fourth, future functional experiments are needed to further uncover the underlying mechanism of pRS-related ceRNA networks as well as the circ_0002951/miR-548k/HAS2 pathway.

## Conclusions

This study established a pRS scoring system based on the comprehensive analysis of the autophagy effects, methylation and immune infiltration on tumor microenvironment, and the prognosis of laryngeal cancer at both macrolevel and microlevel. The differences in pRS were found to be closely related to the SNP, CNV and immunotherapy targets of laryngeal cancer cells. The screened ceRNA network and circ_0002951/miR-548k/HAS2 pathway based on pRS differences may reveal the potential molecular mechanism of laryngeal cancer lethality. Moreover, single-cell RNA-seq and ligand receptor analysis shows that excessive infiltration of fibroblasts is associated with poor prognosis. Finally, a neural network-based deep learning model was also established to predict laryngocarcinoma cell types using pRS-related hub gene signatures. Overall, a comprehensive evaluation of individual tumor autophagy, methylation and immune infiltration enables better understanding of the tumor microenvironment and provides individualized immunotherapy strategies.

## Highlights

- We found that the prognostic risk scoring system (pRS) can be used to comprehensively evaluate the prognosis of patients with laryngeal cancer as well as assess the tumor microenvironment, DNA copy number variation and tumor mutation burden in patients with laryngeal cancer;
- A high correlation between pRS and PD-1 expression may provide insights into devising new drug combination strategies or new immunotherapy drugs;
- The ceRNA network and circ_0002951/miR-548k/HAS2 provide potential therapeutic targets and biomarkers of laryngocarcinoma;
- Single-cell RNA-seq and ligand receptor analysis showed that excessive infiltration of fibroblasts was associated with poor prognosis;
- A neural network-based deep learning model was also established to predict laryngocarcinoma cell types using pRS-related hub gene signatures.

## Supplementary Material

Supplementary figures and tables.

## Figures and Tables

**Table 1:** Clinical characteristics of patients from TCGA and GEO databases.

	TCGA cohort			GEO cohort		
	Alive (n=66)	Dead with tumor (n=30)	Total (n=96)	Alive (n=73)	Dead with tumor (n=34)	Total (n=107)
**Gender**						
Female	7 (10.6%)	8 (26.7%)	15 (15.6%)	NA	NA	NA
Male	59 (89.4%)	22 (73.3%)	81 (84.4%)	NA	NA	NA
**Age**						
Mean (SD)	60.8 (8)	62.8 (9.6)	61.4 (8.5)	62.9 (10.1)	64.1 (10.4)	63.3 (10.1)
Median (min, max)	61 (41,80)	61.5 (45,82)	61 (41,82)	64 (41,82)	63.5 (41,88)	64 (41,88)
**Grade**						
G1	6 (9.09%)	2 (6.67%)	8 (8.33%)	28 (38.4%)	14 (41.2%)	42 (39.3%)
G2	38 (57.58%)	21 (70.0%)	59 (61.46%)	34 (46.6%)	15 (44.1%)	49 (45.8%)
G3	22 (33.33%)	7 (23.3%)	29 (30.21%)	11 ([15.1%)	5 (14.7%)	16 (15.0%)
**Stage**						
Stage I	2 (3.0%)	0 (0.0%)	2 (2.1%)	NA	NA	NA
Stage II	5 (7.6%)	3 (10.0%)	8 (8.3%)	NA	NA	NA
Stage III	9 (13.6%)	4 (13.3%)	13 (13.5%)	NA	NA	NA
Stage IV	50 (75.8%)	23 (76.7%)	73 (76.0%)	NA	NA	NA

**Table 2:** Harrell's concordance indexes of the pRS, stage, and nomogram in different cohorts

Cohort	C-index
pRS	0.74 (0.67-0.80)
Grade (SEER)	0.60 (0.58-0.61)
Grade (TCGA+GEO+Clinical data)	0.56 (0.55-0.57)
Nomogram	0.66 (0.58-0.74)

pRS: prognostic risk score.

## Abbreviations

ARGS: autophagy-related genes
BPs: biological processes
CCs: cellular components
CNV: copy number variation
CS: conditional survival
DCA: decision curve analyses
GEO: The Gene Expression Omnibus
GO: Gene ontology
GSEA: Gene-set enrichment analysis
GSVA: Gene set variation analysis for microarray and RNA-Seq data
HNC: head and neck cancer
ICB: immune checkpoint blockade
KEGG: Kyoto Encyclopedia of Genes and Genomes
K-M: Kruskal-Wallis
LASSO: the least absolute shrinkage and selection operator
LM22: leukocyte signature matrix
MARGs: methylation/autophagy-related genes
MFs: molecular functions
miRNA: micro-RNA
OS: overall survival
pRS: the prognostic Risk of laryngeal cancer Scoring system
qPCR: Quantitative real-time PCR
ROC: the receiver operating characteristic curve
SNP: simple nucleotide variation
TCGA: The Cancer Genome Atlas
TIDE: Tumor Immune Dysfunction and Exclusion
TIIC: tumor cell immune cells
