# Supplementary material for: An integrative microenvironment approach for laryngeal carcinoma: the role of immune/methylation/autophagy signatures on disease clinical prognosis and single-cell genotypes
Source: J Cancer. 2021 May 13;12(14):4148–71. doi: 10.7150/jca.58076 (PMC8176413; doi:10.7150/jca.58076)
Supplement: Supplementary file 1 — Supplementary figures and tables. [file jcav12p4148s1.pdf]

Supplementary Figures and Tables

Supplementary Figure Legends

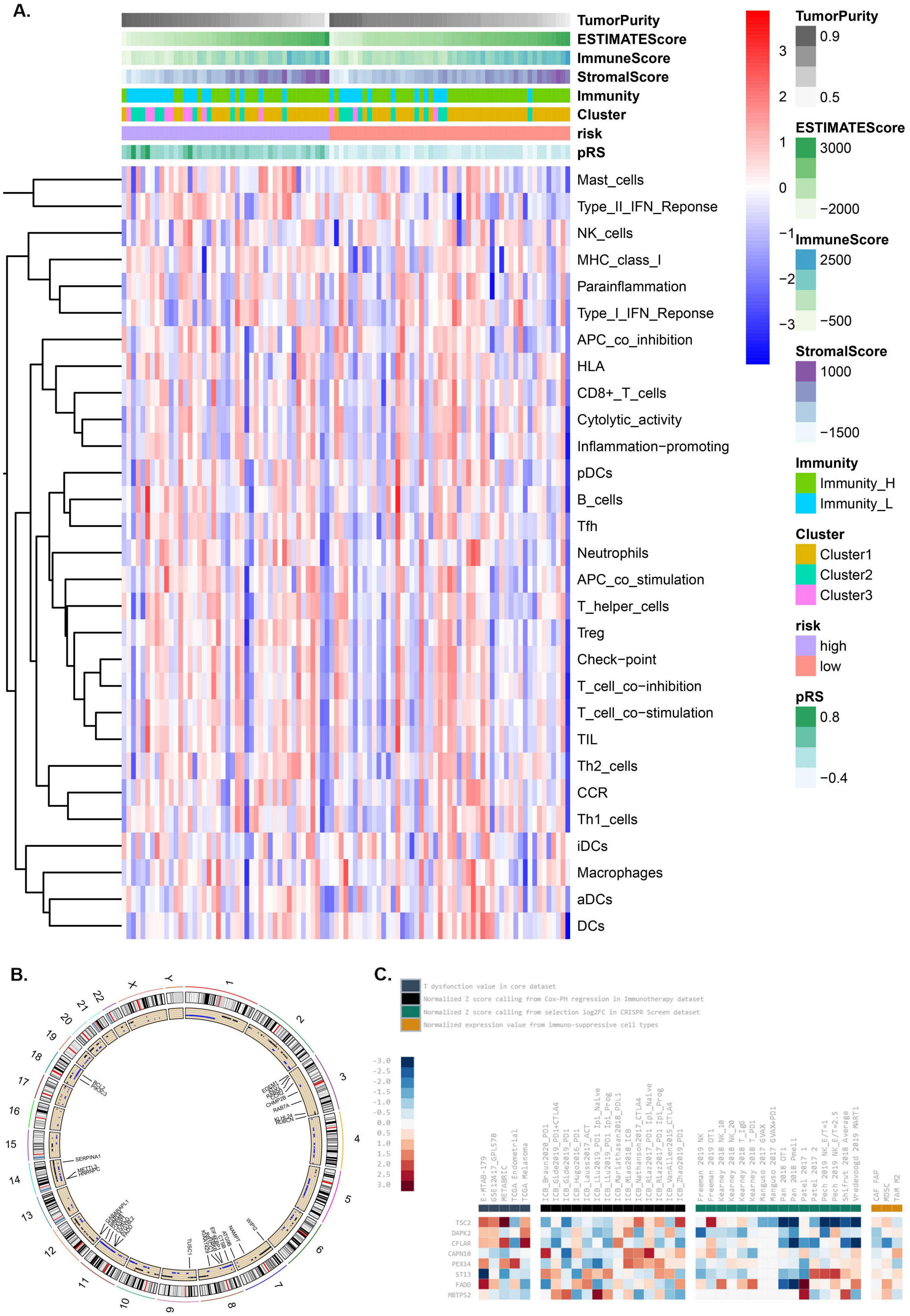

Supplementary Figure 1.

A. A Heat map plot of immune-related functions calculated by ssGSEA.

B. The location of CNV alteration for OS-associated methylated and autophagy associated genes on 23 chromosomes using the TCGA cohort.

C. Heapmap from TIDE used to rank pRS genes (TSC2, DAPK2, CFLAR, CAPN10, PEX14, ST13, FADD and MBTPS2) based on dysfunction and risk score.

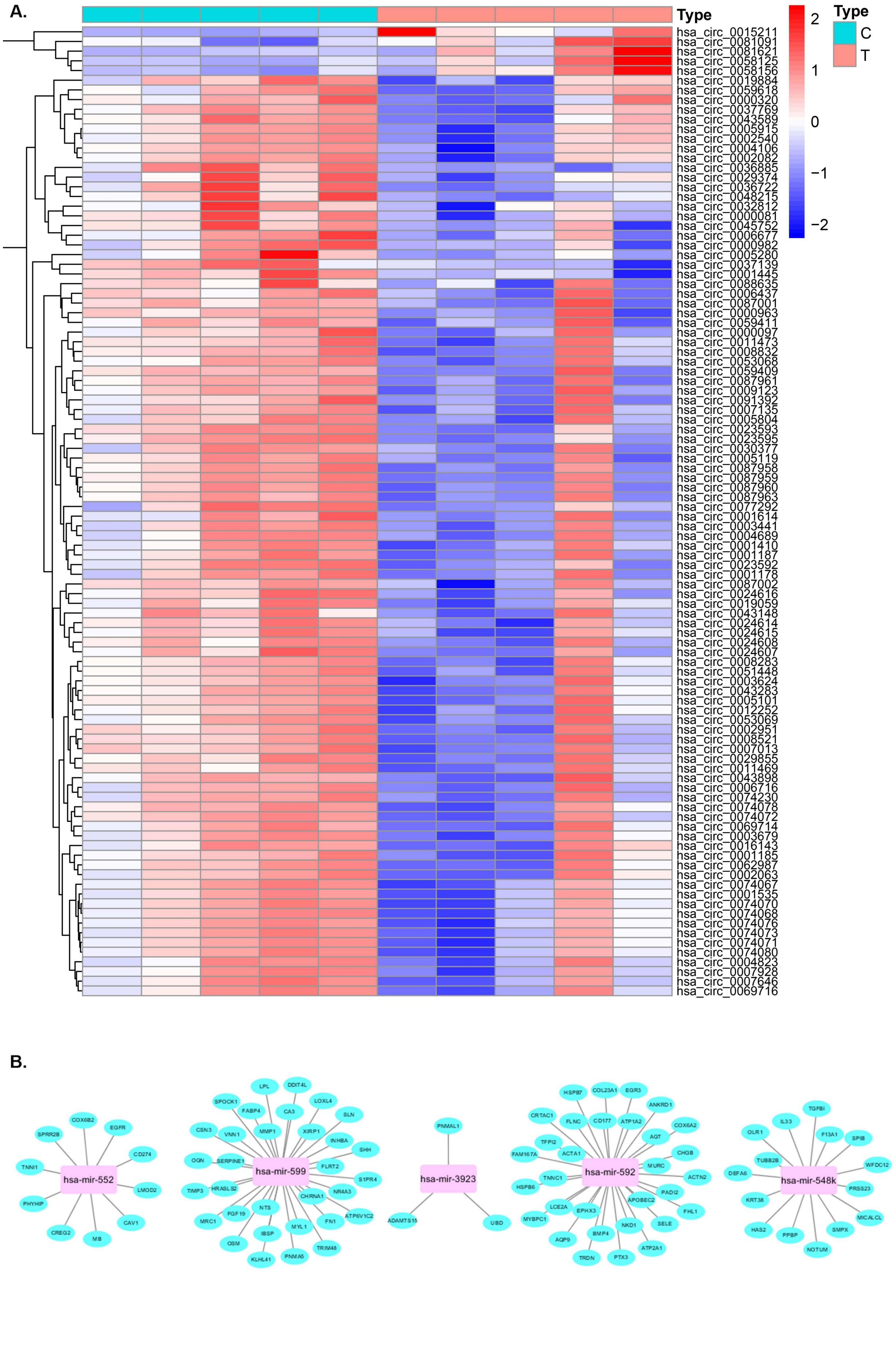
A.
Type
Type
C
T
2
1
0
−1
−2
hsa_circ_0015211
hsa_circ_0081091
hsa_circ_0081621
hsa_circ_0058125
hsa_circ_0058156
hsa_circ_0019884
hsa_circ_0059618
hsa_circ_0000320
hsa_circ_0037769
hsa_circ_0043589
hsa_circ_0005915
hsa_circ_0002540
hsa_circ_0004106
hsa_circ_0002082
hsa_circ_0036885
hsa_circ_0029374
hsa_circ_0036722
hsa_circ_0048215
hsa_circ_0032812
hsa_circ_0000081
hsa_circ_0045752
hsa_circ_0006677
hsa_circ_0000982
hsa_circ_0005280
hsa_circ_0037139
hsa_circ_0001445
hsa_circ_0088635
hsa_circ_0006437
hsa_circ_0087001
hsa_circ_0000963
hsa_circ_0059411
hsa_circ_0000097
hsa_circ_0011473
hsa_circ_0008832
hsa_circ_0053068
hsa_circ_0059409
hsa_circ_0087961
hsa_circ_0009123
hsa_circ_0091392
hsa_circ_0007135
hsa_circ_0005804
hsa_circ_0023593
hsa_circ_0023595
hsa_circ_0030377
hsa_circ_0005119
hsa_circ_0087958
hsa_circ_0087959
hsa_circ_0087960
hsa_circ_0087963
hsa_circ_0077292
hsa_circ_0001614
hsa_circ_0003441
hsa_circ_0004689
hsa_circ_0001410
hsa_circ_0001187
hsa_circ_0023592
hsa_circ_0001178
hsa_circ_0087002
hsa_circ_0024616
hsa_circ_0019059
hsa_circ_0043148
hsa_circ_0024614
hsa_circ_0024615
hsa_circ_0024608
hsa_circ_0024607
hsa_circ_0008283
hsa_circ_0051448
hsa_circ_0003624
hsa_circ_0043283
hsa_circ_0005101
hsa_circ_0012252
hsa_circ_0053069
hsa_circ_0002951
hsa_circ_0008521
hsa_circ_0007013
hsa_circ_0029855
hsa_circ_0011469
hsa_circ_0043898
hsa_circ_0006716
hsa_circ_0074230
hsa_circ_0074078
hsa_circ_0074072
hsa_circ_0069714
hsa_circ_0003679
hsa_circ_0016143
hsa_circ_0001185
hsa_circ_0062987
hsa_circ_0002063
hsa_circ_0074067
hsa_circ_0001535
hsa_circ_0074070
hsa_circ_0074068
hsa_circ_0074076
hsa_circ_0074073
hsa_circ_0074071
hsa_circ_0074080
hsa_circ_0004823
hsa_circ_0007928
hsa_circ_0007646
hsa_circ_0069716
B.
hsa-mir-552
COX6B2
EGFR
SPRR2B
CD274
TNNI1
LMOD2
PHYHIP
CAV1
CREG2
MB
hsa-mir-599
LPL
DDIT4L
SPOCK1
LOXL4
FABP4
CA3
SLN
CSN3
VNN1
MMP1
XIRP1
INHBA
OGN
SERPINE1
SHH
FLRT2
S1PR4
TIMP3
HRASLS2
NR4A3
CHRNA1
FGF19
NTS
ATP6V1C2
MRC1
FN1
IBSP
MYL1
OSM
TRIM48
KLHL41
PNMA5
hsa-mir-3923
PNMAL1
ADAMTS15
UBD
hsa-mir-592
HSPB7
COL23A1
EGR3
ANKRD1
CRTAC1
FLNC
CD177
ATP1A2
COX6A2
TFPI2
AGT
FAM167A
ACTA1
CHGB
MURC
ACTN2
TNNC1
HSPB6
APOBEC2
PADI2
LCE2A
EPHX3
FHL1
MYBPC1
NKD1
SELE
BMP4
AQP9
ATP2A1
TRDN
PTX3
hsa-mir-548k
TGFBI
IL33
OLR1
F13A1
SPIB
TUBB2B
WFDC12
DEFA6
PRSS23
KRT38
MICALCL
HAS2
PPBP
SMPX
NOTUM

Supplementary Figure 2.

A. Heat map of differentially expressed circ-RNAs from GSE117001;

B. The regulatory network between pRS-related mRNAs and pRS-related miRNAs.

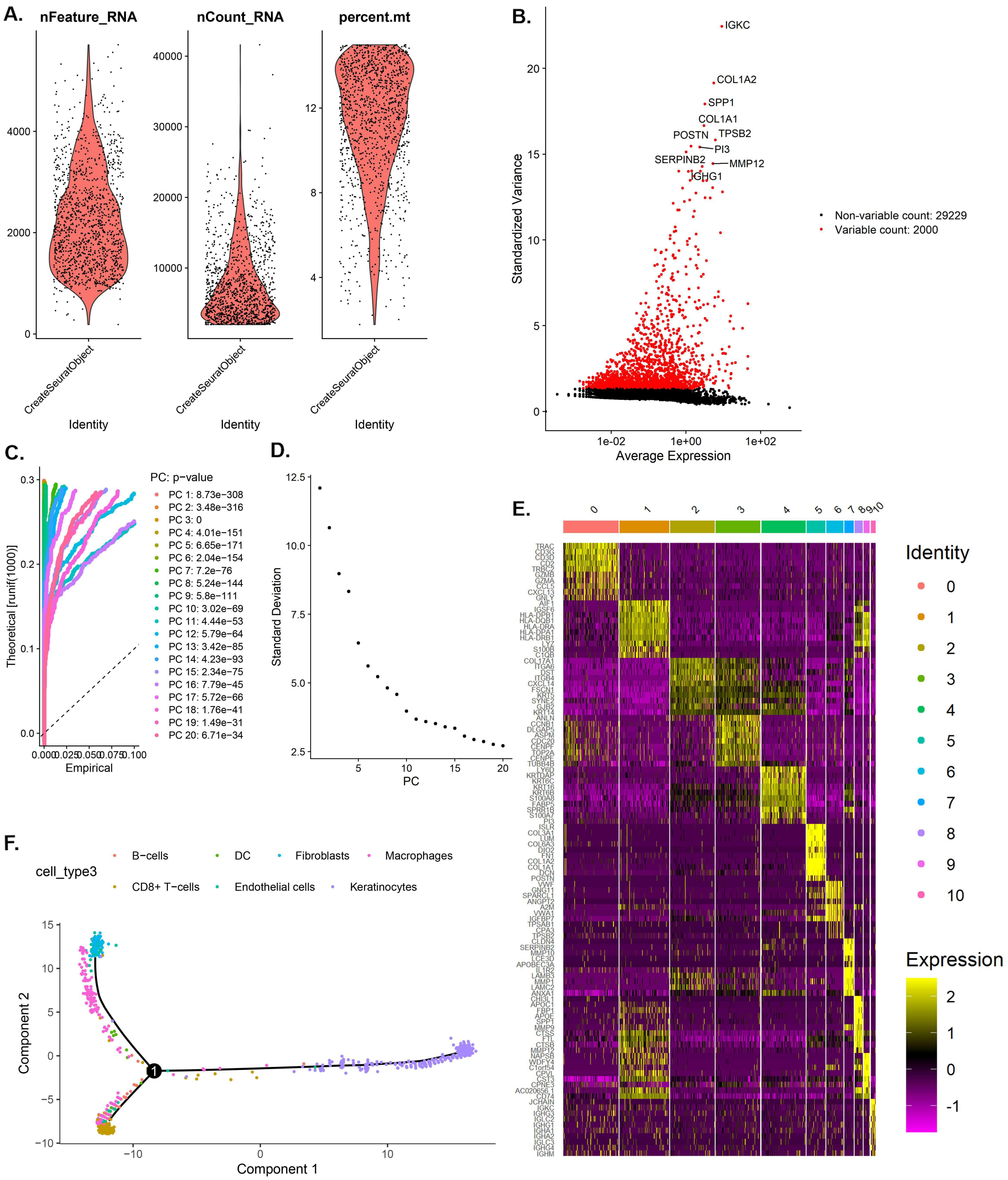
A.
nFeature_RNA
nCount_RNA
percent.mt
CreateSeuratObject
Identity
B.
IGKC
COL1A2
SPP1
COL1A1
POSTN
TPSB2
PI3
SERPINB2
MMP12
IGHG1
Standardized Variance
Average Expression
Non-variable count: 29229
Variable count: 2000
C.
PC: p-value
PC 1: 8.73e−308
PC 2: 3.48e−316
PC 3: 0
PC 4: 4.01e−151
PC 5: 6.65e−171
PC 6: 2.04e−154
PC 7: 7.2e−76
PC 8: 5.24e−144
PC 9: 5.8e−111
PC 10: 3.02e−69
PC 11: 4.44e−53
PC 12: 5.79e−64
PC 13: 3.42e−85
PC 14: 4.23e−93
PC 15: 2.34e−75
PC 16: 7.79e−45
PC 17: 5.72e−66
PC 18: 1.76e−41
PC 19: 1.49e−31
PC 20: 6.71e−34
Theoretical [runif(1000)]
Empirical
D.
Standard Deviation
PC
E.
Identity
Expression
F.
cell_type3
B-cells
DC
Fibroblasts
Macrophages
CD8+ T-cells
Endothelial cells
Keratinocytes
Component 1
Component 2

Supplementary Figure 4. RNA-seq data analysis reveal the high heterogeneity of laryngocarcinoma single cells.

A. 1229 laryngocarcinoma cells were included for analysis;

B. ANOVA plots revealed 2000 corresponding genes in all laryngocarcinoma cells. Red dots indicate highly variable genes and black dots represent non-variable genes. The top 20 marker genes were labeled;

C-D. PCA identified 20 PCs with an estimated P value < 0.05;

E. The top 20 marker genes for each cell cluster were revealed in the heat map.

A.

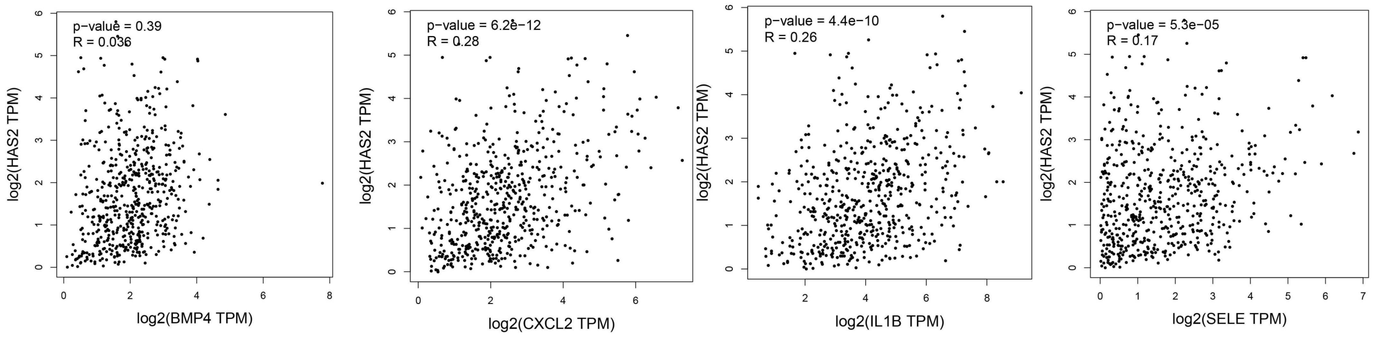
p−value = 0.39
R = 0.036
log2(HAS2 TPM)
log2(BMP4 TPM)
p−value = 6.2e−12
R = 0.28
log2(HAS2 TPM)
log2(CXCL2 TPM)
p−value = 4.4e−10
R = 0.26
log2(HAS2 TPM)
log2(IL1B TPM)
p−value = 5.3e−05
R = 0.17
log2(HAS2 TPM)
log2(SELE TPM)

B.

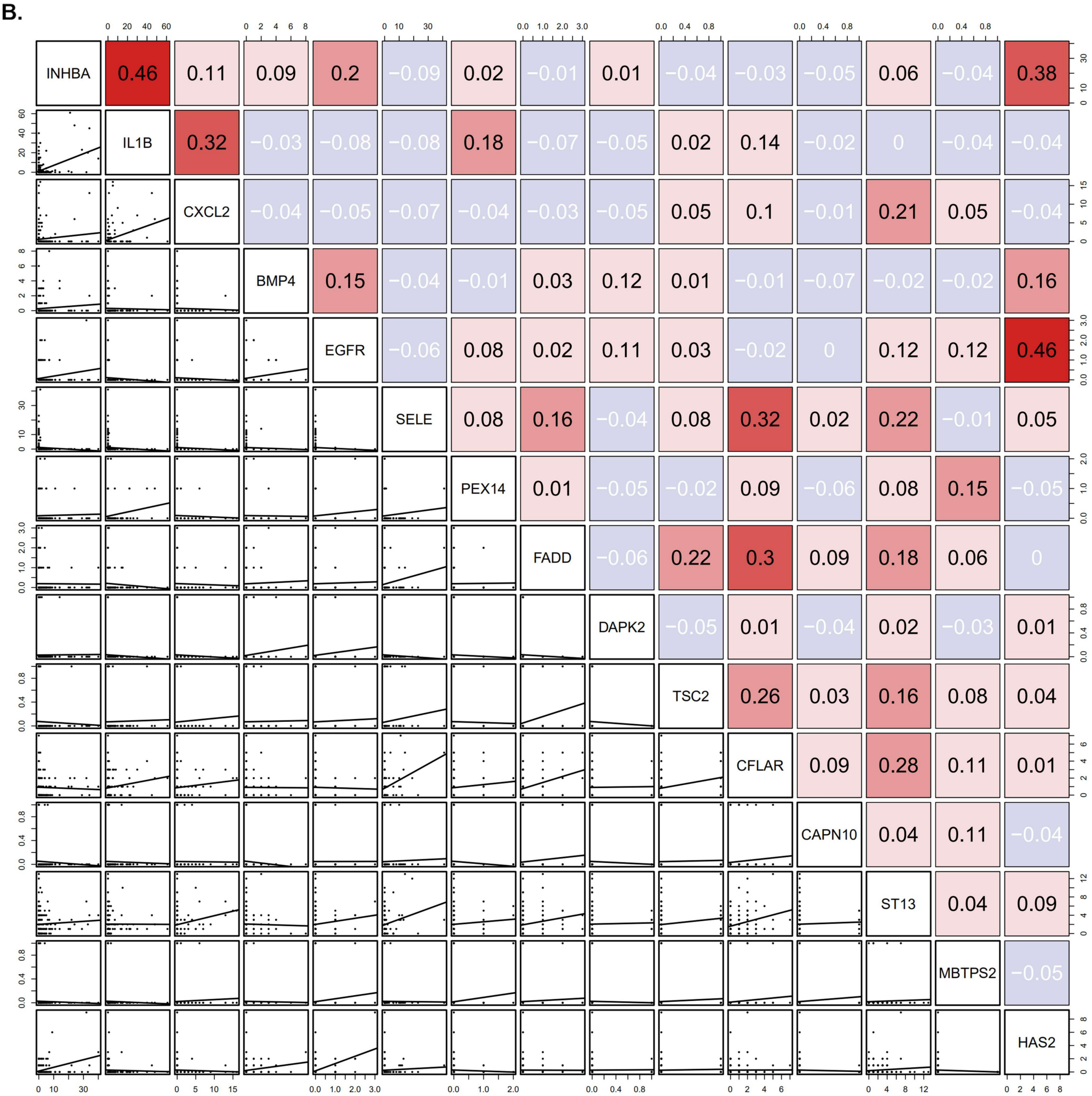
INHBA 0.46 0.11 0.09 0.2 −0.09 0.02 −0.01 0.01 −0.04 −0.03 −0.05 0.06 −0.04 0.38
IL1B 0.32 −0.03 −0.08 −0.08 0.18 −0.07 −0.05 0.02 0.14 −0.02 0 −0.04 −0.04
CXCL2 −0.04 −0.05 −0.07 −0.04 −0.03 −0.05 0.05 0.1 −0.01 0.21 0.05 −0.04
BMP4 0.15 −0.04 −0.01 0.03 0.12 0.01 −0.01 −0.07 −0.02 −0.02 0.16
EGFR −0.06 0.08 0.02 0.11 0.03 −0.02 0 0.12 0.12 0.46
SELE 0.08 0.16 −0.04 0.08 0.32 0.02 0.22 −0.01 0.05
PEX14 0.01 −0.05 −0.02 0.09 −0.06 0.08 0.15 −0.05
FADD −0.06 0.22 0.3 0.09 0.18 0.06 0
DAPK2 −0.05 0.01 −0.04 0.02 −0.03 0.01
TSC2 0.26 0.03 0.16 0.08 0.04
CFLAR 0.09 0.28 0.11 0.01
CAPN10 0.04 0.11 −0.04
ST13 0.04 0.09
MBTPS2 −0.05
HAS2

Supplementary Figure 5.

A. Correlation analysis of HAS2 with BMP4, CXCL2, IL1B and SELE respectively based on TCGA database;

B. Correlations among INHBA, IL-1B, CXCL2, BMP4, EGFR, SELE, PEX14, FADD, DAPK2, TSC2, CFLAR, CAPN10, ST13, MBTPS and HAS2 in the scRNA-seq data.

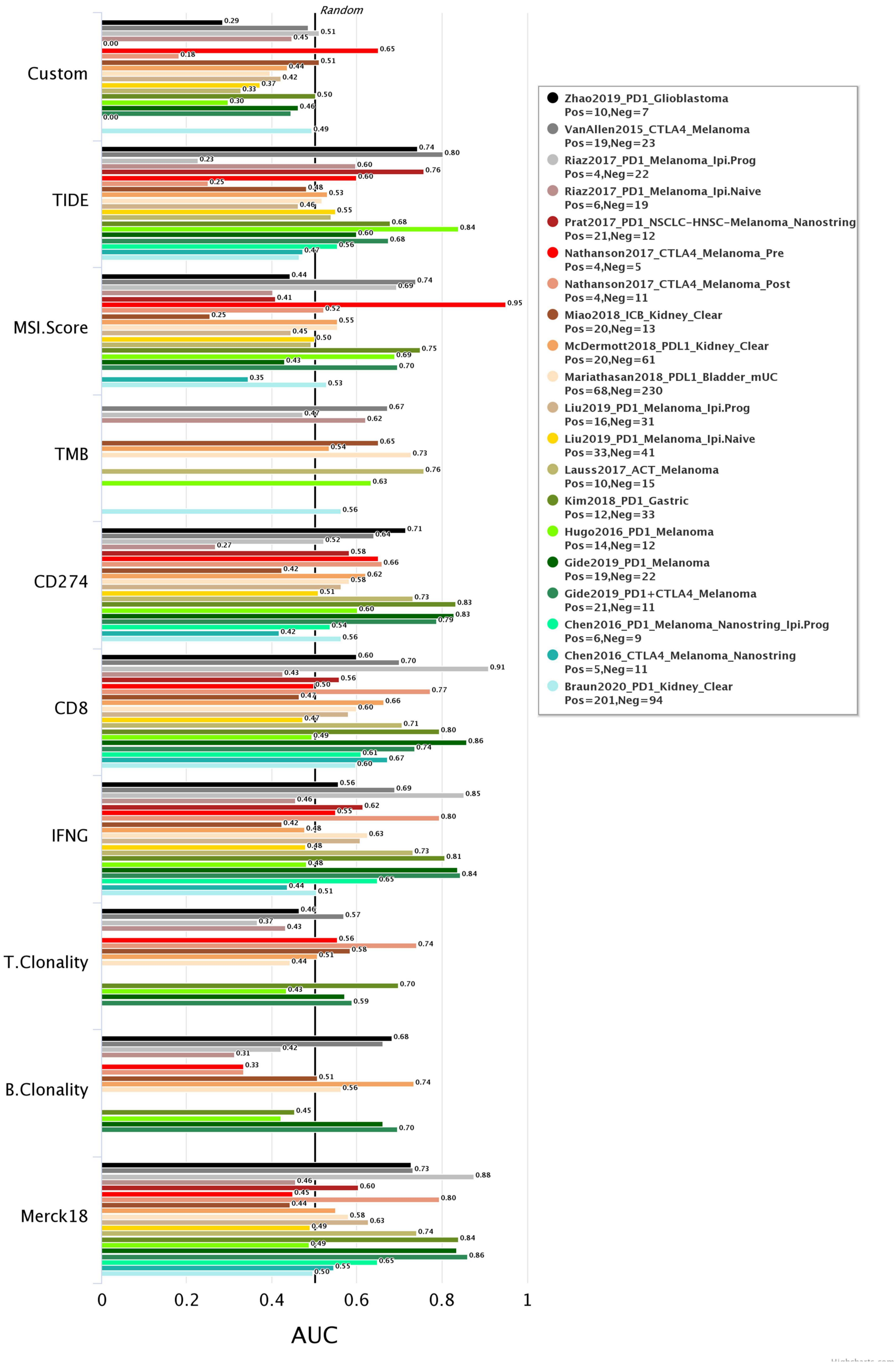

Supplementary Figure 3. An TIDE analysis of compare HAS2 with existed biomarkers.

Supplementary Tables

Supplementary Table 1. Baseline characteristics of 6058 patients from SEER database

	Alive(n=6058)	Dead with tumor(n=3526)	Total (n=9584)	P Value
**Gender**				
Female	1204 (19.9%)	708 (20.1%)	1912 (19.9%)	
Male	4854 (80.1%)	2818 (79.9%)	7672 (80.1%)	0.829
**Age**				
Mean (SD)	62.2 (10.9)	64.5 (10.8)	63 (10.9)	
Median [MIN, MAX]	62 [13,98]	64 [32,98]	63 [13,98]	
**Grade***				
G1	1295 (21.4%)	542 (15.4%)	1837 (19.2%)	
G2	3657 (60.4%)	1893 (53.7%)	5550 (57.9%)	
G3	1058 (17.5%)	1048 (29.7%)	2106 (22.0%)	
G4	48 (0.8%)	43 (1.2%)	91 (0.9%)	1.60E-46
**Stage***				
I	2647 (43.7%)	692 (19.6%)	3339 (34.8%)	
II	1117 (18.4%)	464 (13.2%)	1581 (16.5%)	
III	1020 (16.8%)	798 (22.6%)	1818 (19.0%)	
IV	1274 (21.0%)	1572 (44.6%)	2846 (29.7%)	5.50E-187
**T***				
T0	1 (0.0%)		1 (0.0%)	
T1	2772 (45.8%)	828 (23.5%)	3600 (37.6%)	
T2	1482 (24.5%)	837 (23.7%)	2319 (24.2%)	
T3	1138 (18.8%)	1030 (29.2%)	2168 (22.6%)	
T4	665 (11.0%)	831 (23.6%)	1496 (15.6%)	7.30E-136
**N***				
N0	4787 (79.0%)	1960 (55.6%)	6747 (70.4%)	
N1	464 (7.7%)	458 (13.0%)	922 (9.6%)	
N2	780 (12.9%)	1029 (29.2%)	1809 (18.9%)	
N3	27 (0.4%)	79 (2.2%)	106 (1.1%)	9.40E-134
**M***				
M0	6005 (99.1%)	3319 (94.1%)	9324 (97.3%)	
M1	53 (0.9%)	207 (5.9%)	260 (2.7%)	2.40E-47
**Primary Site***				
Glottis	3872 (63.9%)	1401 (39.7%)	5273 (55.0%)	
Laryngeal cartilage	5 (0.1%)	9 (0.3%)	14 (0.1%)	

Overlapping lesion	138 (2.3%)	167 (4.7%)	305 (3.2%)	
Subglottis	96 (1.6%)	73 (2.1%)	169 (1.8%)	
Supraglottis	1947 (32.1%)	1876 (53.2%)	3823 (39.9%)	2.10E-114

* significant diffierent

Supplementary Table 2. Data sources

Laryngocarcinoma data from authors' hospital	Shanghai ninth people's Hospital
TCGA-HNCS RNA-seq files	Genomic Data Commons
TCGA-HNCS Copy Number Variation data	Genomic Data Commons
TCGA-HNCS Single Nucleotide Polymorphism data	Genomic Data Commons
TCGA gene expression data	Genomic Data Commons
TCGA patients clinic data	Genomic Data Commons
Laryngocarcinoma scRNA-seq data	GSE150321 from GEO database
Laryngocarcinoma RNA-seq files from GEO database	GSE27020 from GEO database
circRNAs data of laryngocarcinoma	GSE117001 from GEO database
